# Purification, Cloning and Immuno-Biochemical Characterization of a Fungal Aspartic Protease Allergen Rhi o 1 from the Airborne Mold *Rhizopus oryzae*

**DOI:** 10.1371/journal.pone.0144547

**Published:** 2015-12-16

**Authors:** Gaurab Sircar, Bodhisattwa Saha, Rahul Shubhra Mandal, Naren Pandey, Sudipto Saha, Swati Gupta Bhattacharya

**Affiliations:** 1 Division of Plant Biology, Bose Institute (Main campus), 93/1 Acharya Prafulla Chandra Road, Kolkata– 700009, West Bengal, India; 2 Biomedical Informatics Center, National Institute of Cholera and Enteric Diseases, Kolkata-700010, West Bengal, India; 3 Department of Allergy and Asthma, Belle Vue Clinic, 9, Dr. U. N. Brahmachari Street, Kolkata—700001, West Bengal, India; 4 Bioinformatics Centre, Bose Institute (Centenary Building), P 1/12, C. I. T. Road, Scheme–VIIM, Kolkata– 700054, West Bengal, India; National Institute of Plant Genome Research, INDIA

## Abstract

**Background:**

Fungal allergy is considered as serious health problem worldwide and is increasing at an alarming rate in the industrialized areas. *Rhizopus oyzae* is a ubiquitously present airborne pathogenic mold and an important source of inhalant allergens for the atopic population of India. Here, we report the biochemical and immunological features of its 44 kDa sero-reactive aspartic protease allergen, which is given the official designation ‘Rhi o 1’.

**Method:**

The natural Rhi o 1 was purified by sequential column chromatography and its amino acid sequence was determined by mass spectrometry and N-terminal sequencing. Based on its amino acid sequence, the cDNA sequence was identified, cloned and expressed to produce recombinant Rhi o 1. The allergenic activity of rRhi o 1 was assessed by means of its IgE reactivity and histamine release ability. The biochemical property of Rhi o 1 was studied by enzyme assay. IgE-inhibition experiments were performed to identify its cross-reactivity with the German cockroach aspartic protease allergen Bla g 2. For precise characterization of the cross-reactive epitope, we used anti-Bla g 2 monoclonal antibodies for their antigenic specificity towards Rhi o 1. A homology based model of Rhi o 1 was built and mapping of the cross-reactive conformational epitope was done using certain *in silico* structural studies.

**Results:**

The purified natural nRhi o 1 was identified as an endopeptidase. The full length allergen cDNA was expressed and purified as recombinant rRhi o 1. Purified rRhi o 1 displayed complete allergenicity similar to the native nRhi o 1. It was recognized by the serum IgE of the selected mold allergy patients and efficiently induced histamine release from the sensitized PBMC cells. This allergen was identified as an active aspartic protease functional in low pH. The Rhi o 1 showed cross reactivity with the cockroach allergen Bla g 2, as it can inhibit IgE binding to rBla g 2 up to certain level. The rBla g 2 was also found to cross-stimulate histamine release from the effector cells sensitized with anti-Rhi o 1 serum IgE. This cross-reactivity was found to be mediated by a common mAb4C3 recognizable conformational epitope. Bioinformatic studies revealed high degree of structural resemblances between the 4C3 binding sites of both the allergens.

**Conclusion/Significance:**

The present study reports for the first time anew fungal aspartic protease allergen designated as Rhi o 1, which triggers IgE-mediated sensitization leading to various allergic diseases. Here we have characterized the recombinant Rhi o 1 and its immunological features including cross-reactive epitope information that will facilitate the component-resolved diagnosis of mold allergy.

## Introduction

The global burden of allergic disorders have reached a pandemic dimensions in which the prevalence of respiratory allergy caused by fungi was estimated to be around 20 to 30% of atopic (allergy-predisposed) individuals or up to 6% of the general population [1]. Mold allergy and asthma have now become a serious health problem worldwide including the urbanized India. School children [2] and people in certain occupations such as farmers, dairymen, loggers, bakers, mill workers, carpenters, greenhouse employees, wine makers and furniture repairers [3] have more exposure to mold and are at greater risk of developing mold allergies. The accuracy of allergy diagnosis is based on the use of purified allergens which is thought to be more accurate and sensitive than using crude antigenic extract. Furthermore, the only disease modifying approach in allergy treatment is thought to be allergen-specific immunotherapy in which the modified hypoallergen is considered to be potential candidate molecule for vaccination in future. Hence, proper identification and molecular characterization of allergens are important in order to efficiently utilize the current diagnostic and therapeutic tools for allergy.

*Rhizopus oryzae* (RO) is a ubiquitously present opportunistic filamentous fungus causing life threatening rhinocerebral and pulmonary mucormycosis infection in immuno-compromised individuals [4]. It is evident from earlier aeromycological study in combination with hospitalization based health survey that this fungus is a major component of fungal aero-spora and causes sensitization to atopic patients leading to allergic disease such as bronchial asthma, atopic rhinitis and dermatitis [5]. This species is currently being biotechnologically exploited for production of industrially important enzymes like tanase, pepsin, laccase and therefore considered as another risk factor for triggering occupational allergic syndrome [6, 7]. In spite of having the clinical reports on the allergenicity of this mold, till now no detailed characterization has been done on the allergenic molecules from this species. This has rendered the clinical diagnosis and therapeutic strategy for the treatment of allergy caused by this mold, quite difficult.

There have been few reports in the literatures that support the presence of IgE antibody specific for RO in the serum of severe allergy patients [8, 9]. In our earlier immunoproteomic study, fourteen IgE reactive proteins were identified from RO, one of the major allergens being a 44 kD aspartyl endopeptidase [9]. Until now three aspartic proteases have been identified as the major allergens from German cockroach (Bla g 2), American cockroach (Per a 2) and *Aspergillus fumigatus* (Asp f 10) [10–12]. Among these three, Bla g 2 has been studied in great details at molecular and structural levels. This insect allergen is a major sensitizer of asthma patients and a recombinant form of this allergen is now successfully used for clinical diagnosis of allergy [13]. Two conformational IgE epitopes have been identified on Bla g 2 and subsequently the hypoallergenic variant of Bla g 2 has also been developed, which holds the potential for being used in allergen immunotherapy [14–15]. It is known from AllFam [16] database that certain other aspartic proteases having also been predicted from various sources such as Japanese cedar, lupine, bovine and pig. In spite of having considerable sequence homology, no in vitro cross reactivity among these aspartic protease allergens has been studied yet. Other than IgE binding property, the protease activity of these allergens is equally important for triggering Th2 response and epithelial barrier disruption [17]. The advancement of recombinant DNA technology and genetic engineering in the field of allergen biology has revolutionized the clinical diagnosis and therapeutic intervention of allergic diseases [18, 19].

In the present study, we describe the biochemical, immunological and structural features of a new 44 kDa aspartic protease allergen from the airborne pathogenic mold *Rhizopus oryzae*. This allergen has been given the official name ‘Rhi o 1’. We have identified and purified this allergen in natural as well as in recombinant form. The present study is also the first report on cross-IgE reactivity between aspartic protease allergens. In fact, to the best of our knowledge, Rhi o 1 is the first major allergen reported from *R*. *oryzae* and as well as from the Zygomycota group of fungi.

## Materials and Methods

### Ethics statement

The present study protocol was approved by the human ethics committee of Bose Institute and Mediland Diagnostic Clinic, Kolkata. Informed written consents were obtained from patients and non-allergic volunteers for participation in the study. In case of minors, informed written consents were obtained from their guardians.

### Fungal culture, chemical, kits and reagents

Pure culture of *Rhizopus oryzae* was maintained in potato dextrose broth (PDB) by repeated sub-culturing in every fifteen days. All the chemicals, kits and reagents were of high purity and purchased from Millipore Corporation (USA), Sigma-Aldrich (USA), Qiagen (Germany), GE Life sciences (USA), Invitrogen (USA), Promega (USA), Merck (Germany), Bio-Rad (USA), Thermo Fischer (USA), Immunotech (France), Amersham (Sweden), New England Biolabs (UK), Roche (USA), Biobharti (India)

### Patient sample collection

We collected residual sera from fungal allergy patients (n = 14) by visiting Mediland Diagnostics, Kolkata, India, with typical case history and indoor symptoms including asthma, rhinitis, skin irritation and conjunctivitis. These patients were Skin Prick Test positive (+3 or +4) to RO antigen and had RO antigen specific IgE titer ≥ 1.0 kU_A_/L in their serum as measured by ImmunoCAP system (Thermo Fisher, Uppsala, Sweden). These RO allergy patients were found to have minor sensitization with some other allergen sources. Smokers and patients with chronic infection (such as worms and parasites) were excluded. Blood from healthy volunteers were collected for negative control. The clinical characteristics of the selected allergy patients and non-allergic volunteers are given in Table 1.

**Table 1:** Clinical characteristics of the mold allergy patients selected for the present study.

Subject no.	Sex	Age	Grade of SPT with RO-Ag	Total IgE IU/ml(Pathozyme kit)	Specific IgE to RO-AgkU_A_/L(ImmunoCAP)	Family historyofallergy	Atopic Symptoms	Sensitivity to Ag’s other than Ro
1	F	28	+3	1525.28	7.80	Y	BA+AR	Af, An, Afl
2	F	32	+4	2228.32	10.20	Y	BA+AR	Pr, Wh, Fo
3	F	19	+3	4011.60	42.80	Y	BA	Cl, Cc, Gr, Dm, Br, Pr
4	M	21	+3	3301.29	18.23	N	BA	Rn, Af, Ao
5	M	18	+4	4230.00	22.30	Y	BA+AR+AD	Gr, Cn, Sf, Pr, Dm, Cc
6	F	22	+4	1689.20	9.36	Y	AR	Br, At, Ao
7	F	21	+3	1827.88	8.86	Y	BA+AR	Cn, Cc, Aa
8	M	26	+3	1873.52	11.20	N	AR+U	Cp, Br
9	M	24	+3	2367.21	390	Y	BA	Af, Dm
10	M	42	+3	2169.24	18.00	Y	AR+AD	Cn, Aa, Fo
11	M	62	+3	3512.33	26.30	Y	AR+AD	Bv, Cn, Ao, Dm, Aa
12	M	16	+3	4193.30	34.90	Y	BA	Aa, Rn, Af, Cp, Pr, Dm, Aa
13	M	48	+4	3248.42	18.60	Y	BA	Af, Afl, Aa, Dm, Pr
14	F	47	+3	2365.26	8.36	Y	BA	Aa, Cl, Ao, Pr
C1	F	40	0	103.33	0.25	N	No	No
C2	F	28	0	70.36	0.12	N	No	No
C3	F	59	0	88.35	0.14	N	No	No
C4	M	28	0	68.89	0.13	N	No	No
C5	M	42	0	78.66	0.21	N	No	No
C6	M	60	0	92.00	0.10	N	No	No

**Footnote:** Here, the grade of SPT positivity is based on the wheal diameter (in mm), where 0 (no wheal), + 1 (2 mm), +2 (3 mm), +3 (4 mm) and +4 (>4.5 mm with pseudopodia).

**Abbreviations:** Ag: antigens; AR: atopic rhinitis; AD: atopic dermatitis Ao: *Aspergillus oryzae*;Afl: *Aspergillus flavus*; At: *Aspergillus tamari*i; Aa: *Alternaria alternata* Af: *Aspergillus fumigatus*; An: *Aspergillus niger*; BA: bronchial asthma; Br: Birch; Cl: *Curvularia lunata*; Cc: *Cladosporium cladosporioides*; Cn: Coconut; Cp: *Carica papaya*; Dm: Dust mite; Fo: *Fusarium oxysporium*; Gr: Grass pollen; N: no; Pr: Prawn; RO: *Rhizopus oryzae*; Rn: *Rhizopus nigricans*; Sf: Sunflower; U: urticaria; Wh: wheat; Y: yes.

### Protein extraction from *R*. *oryzae*

Around 15 grams of densely sporulated mycelial mat of the fungus was harvested from the broth media when the pH of the media dropped to ~3.4. The mat was then lyophilized to dryness and defatted with n-hexane. The mat was homogenized into powder using liquid nitrogen, which was resuspended in 100 ml of 100 mM phosphate buffer saline (pH-7.2) containing 10 mM EDTA, 3 mM sodium azide, 1% glycerol and 10 mM PMSF. The mixture was stirred overnight at 4°C. Next day, the clear extract was obtained after centrifugation, which was brought to 70% ammonium sulphate saturation. Following centrifugation the supernatant was discarded and the protein pellet was reconstituted in 20 mM Bis-Tris buffer (pH-6.5). This pellet fraction was dialyzed extensively against the same buffer overnight at 4°C to remove the traces of ammonium sulphate and finally used for column purification.

### Purification of native 44 kDa nRhi o 1

All the column purification steps were carried out with prepacked columns in AKTAprime plus protein purification system (GE life sciences, Uppsala, Sweden). The proteins present in the pellet fraction with Bis-tris buffer was loaded onto ‘Q’-column. After removal of unbound proteins as flow through, the column was washed thoroughly with the same buffer. The column bound proteins were eluted with 0–1.0 M linear gradient of NaCl. Fraction containing Rhi o 1 was buffer exchanged for 20 mM phosphate buffer (pH-7.0) and loaded onto Superdex^TM^ 75 10/300 gel filtration column. Eluted fraction containing Rhi o 1 was concentrated in Amicon filtration device with MW cut off < 30 kDa, (Millipore, Bedford, MA) and refractionated by a second round of gel filtration chromatography. All the column eluted fractions were screened by IgE-western blot with pooled patient sera to check for the presence of the desired allergen.

### IgE western blot

Proteins were resolved in SDS-PAGE and then transferred onto PVDF membrane. The membranes were blocked with 3% BSA and then confronted with 1:10 diluted RO positive patient sera for overnight at 4°C. After washing, the bound IgE was detected with 1:1000 diluted AP conjugated anti-human IgE (Sigma) and blots were developed with NBT-BCIP. For negative control, the pool serum of all the six healthy individuals was used for western blot.

### IgE-ELISA

ELISA plate bound purified allergen (5ng/μl) was blocked with 0.5% BSA in 50 mM PBS-Tween-20 (pH 7.2). Plates were then exposed to 1:10 diluted serum of RO positive patients (or, healthy sera as negative control) at 4°C for overnight. Bound IgE was detected with 1:1000 diluted AP-tagged monoclonal anti-human IgE (Sigma) for 3 hrs at 4°C. Color was developed with pNPP substrate (Sigma) and OD was taken at 405 nm. All the reactions were done in four replicates. The average OD_405_ value of all the replicates for each patient was calculated as ‘P’ and for all the six normal subjects was calculated as ‘N’. The ratio between mean OD_405_ values of each patient (P) and healthy (N) sera was calculated as described previously [8]. For a particular patient, P/N ≥ 3.5 was considered as having markedly elevated level of allergen specific IgE.

### Mass spectrometry and N-terminal sequencing

The purified native Rhi o 1 was isoelectrofocused in 2D gel using IPG strip, pH 4–7, (GE Healthcare, Uppsala, Sweden). The desired spot was excised and prepared for MALDI analysis as described by Shevchenko et al. [20]. Briefly, the spot was destained in 50 mM Ammonium bicarbonate (pH 8.0) with 50% ethanol followed by periodic dehydration and rehydration in Acetonitrile and reduction buffer containing DTT as well as alkylation buffer containing Iodoacetamide (Sigma, St Louis, MO, USA). Digestion was carried out in 12.5 ng/μl modified sequencing grade Trypsin Gold (Promega, Madison, WI, USA) at 37°C for 16 h. Tryptic fragments were eluted from gel pieces by vigorous vortexing in extraction buffer containing 1% TFA. Final volume of the sample was reduced 10 times to remove Acetonitrile in Speed Vac (Thermo Fisher, USA). 1.5 μl of peptide digests were mixed with 5 volumes of 0.5 mg/ml α-cyano-4-hydroxycinnamic acid (HCCA) matrix solution (Bruker Daltonics, Germany), was spotted on MTP 384 ground steel target plate (Bruker Daltonics, Germany) and run in Autoflex-II MALDI-TOF/TOF mass spectrometer (Bruker, Daltonics, Germany). The MS spectra of the peptides and MS/MS spectra of the peptide fragments were analyzed in MASCOT (http://www.matrixscience.com) search engine version 2.2 (Matrix Science, Boston, MA, USA) for protein/peptide identification. For N-terminal sequence analysis, the purified Rhi o 1 was transferred from gel to PVDF membrane using 10 mM CAPS buffer (pH 11) containing 10% methanol and then microsequenced in a Procise protein sequencer (Applied Biosystems, Weiterstadt, Germany).

### cDNA Cloning, over-expression and purification of recombinant rRhi o 1

The amino acid sequence of the allergen was searched against RO transcriptome database at Broad Institute server [21] using tBLASTn tool to identify the transcript. The transcript was then searched against the RO genome database in NCBI to identify the full length gene. The transcript and full length gene (with introns) of the allergen were deposited in NCBI. The mature ORF was PCR amplified from the RO cDNA library (a kind gift from Dr. Christopher D. Skory of National Center for Agricultural Research Utilization, USDA-ARS) using forward (5′GCGCGGATCCATGAAATTTTTTGCATTATCTCT3′) and reverse (5′AATAAAGCTTTTATTTGGAAGGAGCCAAACCAA3′) primers containing BamHI and HindIII sites (underlined) respectively. The amplified cDNA was cloned in pRSETA vector (Invitrogen) and transformed into *E*. *coli* DH5α strains. Positive transformants were selected on LB agar plates supplemented with ampicilin at 100 μg/ml concentration. The orientation of the reading frame of the insert was checked by DNA sequencing. Expression of recombinant protein was induced in *E*. *coli* BL21(DE3)Rosetta cells in 1 Liter Luria broth (LB)containing ampicilin (100 μg/ml) and chloramphenicol (34 μg/ml) with 0.5 mM IPTG for 14 hours at 20°C. N-terminal 6x-His tagged recombinant allergen was purified under native condition using Ni-NTA column (Qiagen) following manufacturer’s protocol. Column bound protein was washed twice with 0.1% Triton X-114 and removal of LPS was confirmed by *Limulus amebocyte lysate* assay. Purity and integrity of the recombinant protein was checked in 12% SDS-PAGE and also in mass spectrometry. For further analysis, this Ni-NTA purified rRhi o 1 was again passed through Superdex^TM^ 75 10/300 gel filtration column to achieve higher purity of the recombinant protein. In order to test for the immuno-reactivity, the purified recombinant allergen was confronted with ten individual RO positive allergy patient sera in IgE-western blot. For negative control, pool of six non-allergic healthy sera was used to probe the allergen.

### Stripped basophil Histamine release assay by passive IgE sensitization

Histamine release upon rRhi o 1 challenge was performed and percentage of histamine release was calculated as described previously [22]. Briefly, PBMC’s (2 x 10^5^) were isolated from healthy donors by HiSep^TM^LSM 1077 (HiMedia Laboratories, India) and bound IgE’s were stripped off by incubating in lactic acid buffer (pH 3.5) for 3 min. Stripped basophils were then passively sensitized with serum IgE of ten RO positive patients at 37°C for 120 mins. These IgE-sensitized PBMC’s were then recovered in HEPES buffer and stimulated in triplicate with 30 ng of rRhi o 1. Allergen concentrations used for in vitro stimulations had initially been optimized in titration experiments (data not shown). Histamine released in the cell-free supernatant was estimated by EIA Histamine assay kit (Immunotech, Beckman Coulter).

### Enzyme assay for aspartic protease

Functional activity of the purified Rhi o 1 was studied by:

1. Gelatin Zymography: Rhi o 1 was resolved in 7% non-reducing SDS-PAGE containing 0.02% gelatin as described [23] and the gel was washed twice in 10 mM Tris (pH 7.5) containing 0.25M NaCl and 2% Triton-X. The gel was then incubated in 0.1 (M) Tris-glycine buffer (pH 7.5) for overnight at 37°C. Protease activity of Rhi o 1 was visualized by coomassie staining of the gel.
2. Spectrophotometric assay with BSA as substrate: In this in-sol assay, each reaction set contained 600 μl of 2% BSA in 50 mM sodium citrate (pH 3.2) and 100 nM of Rhi o 1 as previously described [24]. Reactions were incubated at 37°C from 0 to 360 seconds with 30 seconds interval and stopped by adding 2(M) perchloric acid. Precipitated proteins were removed by centrifugation and protease activity was measured by recording the increase in A_280_ of the clear supernatant.

### Circular Dichroism (CD) spectroscopy

CD spectra of around 0.142 mg/ml of nRhi o 1 and rRhi o 1 were recorded in Jasco Corp. J-815 CD spectropolarimeter at 20°C within a wavelength range of 260–200 nm. Average spectra of five scans were analyzed in K2D of Dichroweb server [25].

### Multiple sequence alignment and phylogenetic tree

Rhi o 1 sequence was compared with eleven characterized/predicted aspartic protease allergens reported in AllFam database [15] using T-coffee alignment tool. A phylogenetic tree was constructed for these twelve aspartic protease allergens by Maximum Likelihood method with 1000 bootstraps using MEGA 6.0 software [26]. A second multiple sequence alignment was also performed with four reported aspartic protease allergens including Rhi o 1 using T-coffee alignment tool.

### IgE ELISA inhibition and blot inhibition experiment with Bla g 2

Ample sequence similarity between Rhi o 1 and the cockroach allergen Bla g 2, prompted us to investigate for the presence of cross-reactivity between these two allergens. Recombinant Bla g 2 was kindly gifted by Dr. Martin D. Chapman and Dr. Anna Pomes and Dr. Jill Glesner of Indoor Biotechnology Inc., Charlottesville, Virginia, USA. All ten rRhi o 1 positive serum samples were tested for their reactivity against rBla g 2 by IgE ELISA and P/N ratios were calculated as described previously. The IgE binding of these sera to rBla g 2 was compared with that of the rRhi o 1. Eight such serum samples with substantially high specific IgE titer against Bla g 2, were pooled and mixed separately with serially diluted concentrations of rRhi o 1 (auto inhibitor, +ve control), Bla g 2 (fluid phase inhibitor) and BSA (non-inhibitor, -ve control) respectively at 4°C for overnight. Plate bound 1 μg of rRhi o 1 (solid phase) was separately probed with these preincubated sera. The percentage of IgE ELISA-inhibition was determined by the following formula–

$$1-\left(\frac{\text{OD}405\;\text{of sample with inhibitor}}{\text{OD}405\;\text{of sample without inhibitor}}\right)\times 100$$

In a reciprocal experiment, we coated 1 μg of rBla g 2 on ELISA plate and it was then probed with same patient sera separately mixed with serially diluted concentrations of rRhi o 1 (fluid phase inhibitor), rBla g 2 (auto inhibitor, +ve control) and BSA (non-inhibitor, -ve control) respectively and percentage of IgE inhibition was calculated as described in the earlier experiment.

### IgE-western blot inhibition under denaturing conditions

For IgE-blot inhibition, 5 μg of rRhi o 1 transferred from SDS-PAGE onto PVDF membrane was confronted with rBla g 2 (0.1 to 10 μg) preincubated sera and bound IgE was detected as described in IgE western blot. For positive control, the sera were presaturated with 5μg of rRhi o 1 and auto inhibition was performed in the blot.

For further confirmation of IgE binding to denatured rRhi o 1, we performed IgE-immunodot blot of 0.4 μg of rRhi o 1 heat denatured at 95°C for 10 mins. The denatured rRhi o along with equal amount of non denatured rRhi o 1 (as positive control) were dotted onto PVDF membrane and then exposed to three patient sera in 1:10 dilution. IgE binding to heat denatured rRhi o 1 was tested with monoclonal anti-human IgE with AP conjugate (Sigma) in 1:1000 dilution as described earlier in immunoblot section.

### rBla g 2 stimulated histamine release from PBMC passively sensitized with anti-Rhi o 1 IgE

PBMC’s from healthy donors were passively sensitized with eight Rhi o 1 allergic sera, which were found to also have specific IgE reactivity with rBla g 2. The passively sensitized PBMC’s were then cross-stimulated with 30 ng of rBla g 2 and histamine release was estimated as described earlier.

### Cross reactive IgE epitope identification using murine anti-Bla g 2 mAb’s 4C3, 2F1, 1F3 and 7C11

All the four mAbs developed against the surface epitopes of Bla g 2 were kindly gifted by Dr. Anna Pomes and Dr. Jill Glesner of Indoor Biotechnology Inc., Charlottesville, Virginia, USA. In the first step, each of these four mAb’s was tested for their reactivity against Rhi o 1 by sandwich ELISA as described [27]. Briefly, increasing concentration of rRhi o 1 was added to four plate bound anti-Bla g 2 mAb’s (1μg/ml) for capture. Anti-aspartyl protease pAb from rabbit was used as detection Ab (1:100) and AP-conjugated anti-rabbit IgG (1:1000) was used as secondary Ab. Colour was developed by adding pNPP substrate (Sigma) and OD was taken at 405 nm. In the second step, effect of heat denaturation of rRhi o 1 on mAb4C3 binding was tested by sandwich ELISA. Briefly, around 100 ng/ml rRhi o 1 was incubated in gradually increasing temperatures (25°C to 95°C with 10°C intervals) and coated in ELISA plates. Plate bound heat denatured rRhi o 1 was then exposed to mAb4C3 at 1μg/ml concentration and subsequent steps of ELISA were similar to previous sandwich ELISA experiment.

In the third step, we performed competitive inhibition assay [15] to understand whether mAb4C3 can inhibit serum IgE binding to Rhi o 1. The plate bound 1 μg Rhi o 1 was probed with mAb 4C3 at increasing concentrations of 0, 0.001, 0.01, 0.1, 1 and 10 μg/ml at 4°C for 4 hrs. This was followed by addition of cross-reactive patient sera in 1:10 dilution for 12 hrs at 4°C. A rabbit anti-aspartyl protease polyclonal antibody (Bioorbyt, SF, USA) (1:10 dilutions) instead of 4C3, was used as positive control and anti-6x His polyclonal antibody was used as negative control. For uninhibited control reaction, plate bound rRhi o 1 was incubated with serum IgE only without any inhibitors. Bound IgE’s were detected by AP conjugated anti-human IgE mAb. Percentage of IgE inhibition was calculated using the following formula–

$$1-\left(\frac{\text{OD}405\;\text{of the reaction with serum IgE}+\text{inhibitors}\;(4\text{C}3\;\text{or polyclonal Ab's})}{\text{OD}405\;\text{of the reaction with serum IgE only without inhibitor}}\right)\times 100$$

### Homology modelling, cross-reactive epitope mapping and molecular docking

The homology model for Rhi o 1 was built in SWISS-MODEL Version 8.05 [28] server using yeast aspartic protease (PDB Code 1DPJ) as template and visualized in Pymol. Stereochemical quality of the model was checked in SAVES_3 server [29–32]. The atomic co-ordinates of Bla g 2 crystal structure (PDB Id: 1YG9) were aligned with that of Rhi o 1 to calculate the R.M.S.D in C_α_ backbones. The regions comprising the 4C3 conformational epitope of Bla g 2 was compared with the Rhi o 1 model in order to identify the possible cross IgE reactive region. For molecular docking, the antibody was extracted from the crystal structure of Bla g 2:Fab 4C3 complex (PDB Id: 3LIZ) and was used as a receptor whereas the Rhi o 1 model was set as a ligand for the docking in ClusPro 2.0 server [33]. Interactions were visualized in Discovery Studio 2.5 software.

## Results

### Purification of the native nRhi o 1

**Fig 1:**
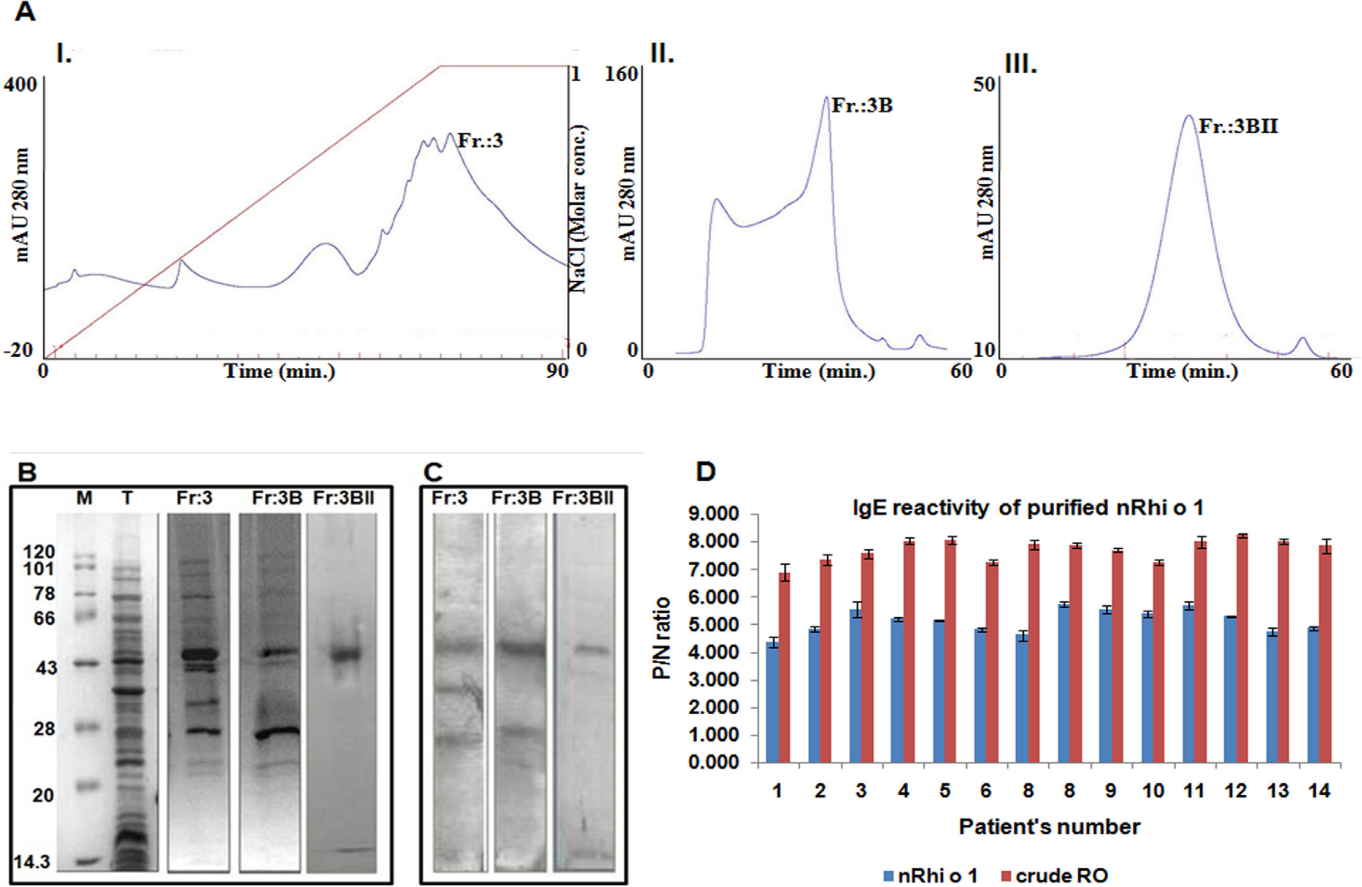
Purification of native nRhi o 1. (A) I. Chromatogram of anion exchange chromatography (Q-HP) of the fraction obtained from 60% ammonium sulfate cut of the total protein extracts. Third fraction (Fr:3) contains the 44 kD Rhi o 1. II. Chromatogram of the first round gel filtration chromatography (Superdex^TM^ 75) with Fr:3. Second fraction (Fr:3B) contains the desired allergen mixed with low molecular weight proteins. III. Chromatogram of the second round of gel filtration chromatography with Fr:3B. Peak (Fr:3BII) represents native Rhi o 1 with desired purity. (B) SDS-PAGE of all the Rhi o 1 containing fractions obtained during three round of column chromatography. Lane: M is molecular wt. marker and Lane: T is fraction obtained from 60% ammonium sulfate cut of the total protein. (C) Screening of these three fractions by IgE-immunoblot with RO positive patient sera to check for the presence of the allergen. Final fraction Fr:3BII showed the presence of a single IgE reactive band at 44 kDa implying the homogeneity of the purified allergen. (D) Specific IgE reactivity of the purified nRhi o1 by ELISA using individual sera from fourteen RO positive patients. ‘Y’ axis represents the P/N ratio, which was observed to be > 4.0for nRhi o 1 (blue bars). This was compared with P/N of the crude allergen extract of RO as positive control (red bars).

Using a combination of ion exchange and gel filtration chromatography, the 44 kD Rhi o 1 was purified in its native form from the spore mycelia of RO. The fraction obtained after 60% ammonium sulphate cut of the total protein extracted from the fungal tissue was loaded onto Q-column. Fig 1AI shows the chromatogram of this anion exchange chromatography in which the third fraction (Fr: 3) was eluted with nearly 80% NaCl gradient and was found to contain the 44 kD allergen. Fr: 3 was reloaded in Superdex^TM^ 75 column and elutes from this column separated into two fractions (Fr: 3A and 3B) as shown in Fig 1AII. The second fraction Fr: 3B contained the 44 kD IgE reactive protein albeit mixed with certain other low molecular weight proteins. In order to achieve substantial purity, a second round of gel filtration chromatography was performed as a final polishing stage. Fr: 3B was concentrated in Amicon Ultra with 30 kD cut-off filter to remove unwanted proteins and then subjected to size exclusion chromatography in the same column. A sharp peak (Fig 1AIII), designated as Fr: 3BII, was observed after 35 ml elution in which the desired Rhi o 1 was present at approximately 90% homogeneity. The IgE reactivity of all these fractions was tested by IgE immunoblot with pooled RO sensitive patient’s sera, as shown in Fig 1B and 1C. The final fraction 3BIIappeared as a single IgE reactive band in the immunoblot (last lane of Fig 1C) implying the desired level of purity and quality of the purified natural Rhi o 1.Purified native Rhi o 1 displayed substantial IgE reactivity as checked by IgE-ELISA with fourteen individual RO allergic sera and six healthy sera (for negative control) as shown in Fig 1D. The P/N ratio for native Rhi o 1 was within a range between 4.20 and 5.90. This was in line with the P/N ratios of RO crude extract, used as positive control, for which the values were between 5.10 and 6.72.

### Identification of nRhi o 1 by mass spectrometry and N-terminal sequencing

**Fig 2:**
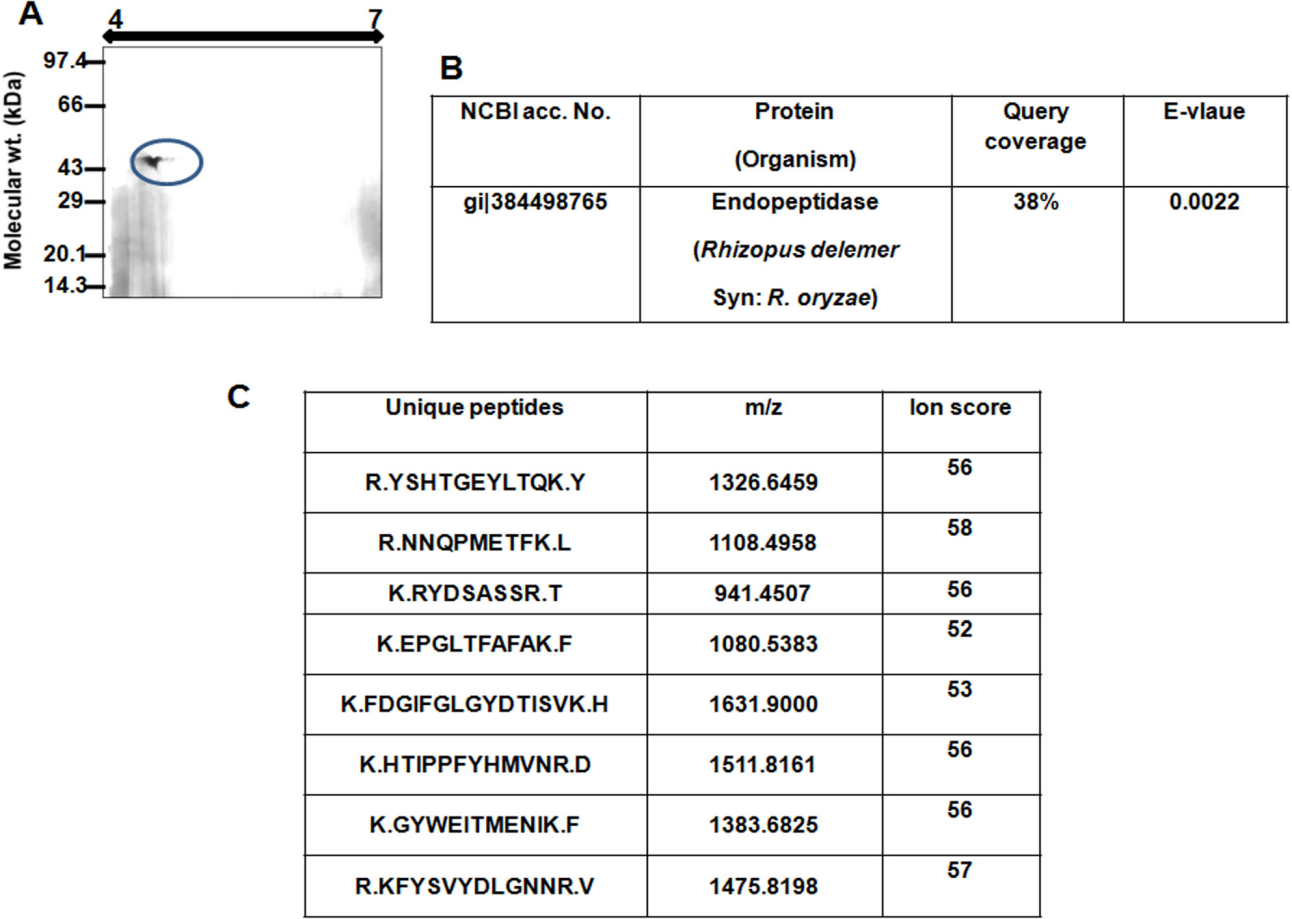
Identification of Rhi o 1. (A) Purified nRhi o 1 was resolved in 2D gel and the observed pI was same with the theoretical pI of the allergen. Single spot on 2D gel suggests the absence of any isoform of Rhi o 1. (B) Results of peptide mass fingerprint of trypsin digested Rhi o 1. MS spectra showed homology with Endopeptidase of RO (gi|384498765) in NCBI database. (C) Mass spectra of Rhi o 1 generated in MALDI-TOF. The box represents the results of MALDI-TOF/TOF of eight unique matched peptides and their corresponding ion scores. MS/MS of all the peptides displayed significant match with the same protein gi|384498765, where the significance level was p > 0.05.

In two-dimensional gel the purified allergen appeared as a single spot with an isoelectric point (pI) at 4.6 and molecular weight of 44 kDa (Fig 2A). There were no other spots in the 2D gel suggesting the purity as well as absence of any isoforms of this allergen. Mass spectrometric identification of the allergen is illustrated in Fig 2B and 2C. The raw MS spectra of the peptides generated by trypsinization were analysed in MASCOT search engine and the allergen was identified as a 401 amino acid long Endopeptidase of RO (gi|38449876). Twelve unique matched peptides were detected that comprised up to 38% sequence coverage. Theoretical pI of this protein was 4.7 and this was in line with what was observed in 2D gel. For reconfirmation, theMS/MS spectra of eight of these twelve unique peptides were analysed in MASCOT, which also displayed identity with Endopeptidase of RO (gi|38449876) with significant ion scores. Ion score is the absolute probability that the observed peptide match is a random event and it is expressed as [-10 X log_10_ (P)], where ‘P’ stands for absolute probability. Amino acid sequence of this protein was retrieved from NCBI protein database and used for cDNA analysis. Edman sequencing of the purified allergen generated the sequence AITKI(?), which represented an N-terminal stretch from 21^st^ to 25^th^ amino acid of Rhi o 1.

### Cloning, expression and purification of recombinant rRhi o 1

**Fig 3:**
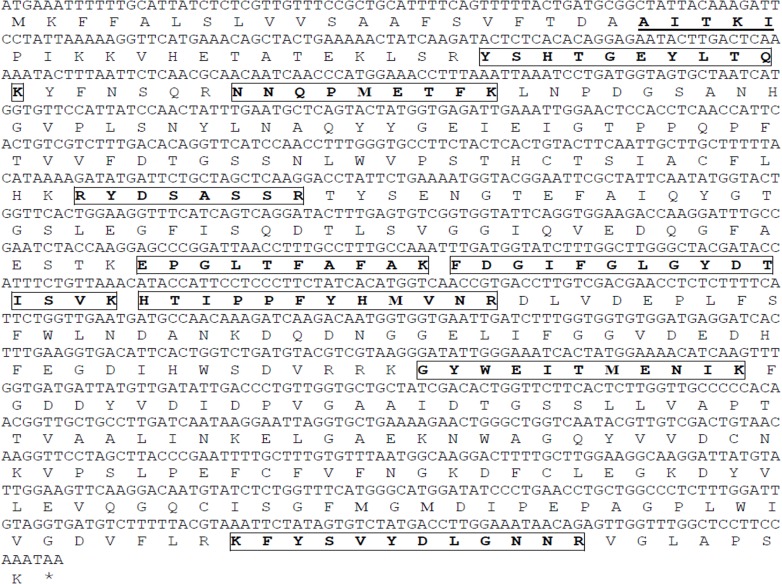
Full length cDNA and amino acid sequence of Rhi o 1. Amino acids, which are bold and underlined, represent those identified by N-terminal sequencing of nRhi o 1 suggesting the presence of an N terminal 20 amino acid long signal peptide. Amino acids shown as bold and within boxes, are those identified by MS/MS.

Upon tBLASTn search with the amino acid against *Rhizopus oryzae* genome database at the Broad Institute server, a 1206 bp long full length transcript was identified under the accession number RO3G_13967.3. The nBLAST analysis with this transcript against RO genome in NCBI showed hit with a 1,38,388 bp long contig (accession no. gbIJNDY01003708) constructed upon shotgun assembly. In this contig, based on the transcript alignment position, a 1398 bp long full length gene could be identified with three introns. We deposited the full length gene and the cDNA sequences of Rhi o 1 in NCBI under the accession numbers KM459699 and KM459700 respectively. The amino acid sequence of Rhi o 1 and its corresponding cDNA sequences reported in this study are shown in Fig 3. This ORF was amplified by RT-PCR from either the cDNA pool of the fungal culture from which nRhi o 1 was purified or the cDNA library of *R*. *oryzae* (RA 99–880), which was a clinical isolate from the brain abscess of mucormycosis patient used in RO genome project. The nucleotide sequence of both the ORF’s were found to be identical suggesting that identification of the allergen by mass spectrometry using NCBInr database was accurate.

**Fig 4:**
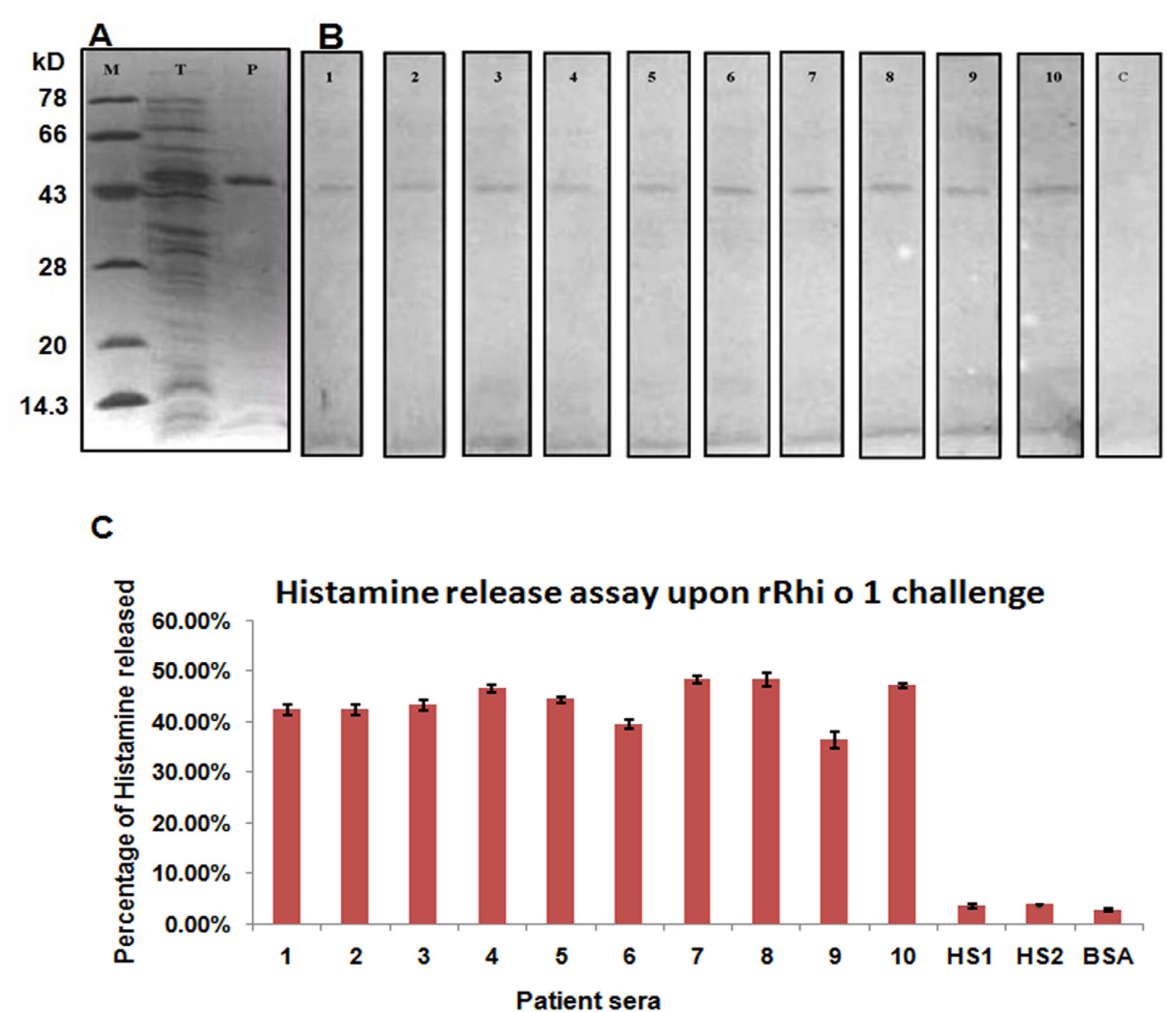
Recombinant rRhi o 1 and its allergenecity. (A) Recombinant expression of Rhi o 1 in *E*. *coli*induced by IPTG. Lane: M is the molecular wt marker; Lane: T is the total protein after 0.5 mM IPTG induction of *E*. *coli* harboring pRSETA-Rhi o 1 cDNA construct and Lane: P is the Ni-NTA purified rRhi o 1. (B) IgE-western blots of rRhi o 1 with individual serum of ten RO positive patients (lane 1–10), which shows the presence of IgE reactivity in rRhi o 1. Lane-c represents the negative control blot with pool of sera from six healthy subjects in which no IgE-reactive bands are visible. (C) Mediator (histamine) release efficiency of rRhi o 1 from effector cells through passive sensitization with sera from ten allergy patients (1–10). HS1 and HS2 represent sensitization with two non-atopic healthy sera as negative control showing minimum percentage of histamine release. Challenge with BSA instead of Rhi o 1 also served as negative control with no histamine release.

The cDNA was expressed in *E*. *coli* and the recombinant protein was purified to homogeneity following the endotoxin removal. The yield of the affinity purification was around 20–25 mgL^-1^ of culture. Fig 4A illustrates the purified recombinant protein without any contaminating band as checked by SDS-PAGE. The molecular weight and sequence integrity of the recombinant protein was verified via MALDI-TOF and was identical to its native counterpart.

### Allergenic activity of rRhi o 1

The allergenic activity of the recombinant protein was tested in terms of its IgE reactivity and mediator release efficiency. The rRhi o 1 was probed with ten individual *R*. *oryzae* sensitive sera in immunoblot. As shown in Fig 4B, the rRhi o1 was found to be recognized by specific IgE antibody in the serum of all the ten allergy patients. The IgE-immunoblots of the remaining four atopic subjects could not be done because of unavailability of their sera. In case of negative control blot, no IgE reactive bands were observed.

In addition to IgE reactivity, the inflammatory mediator release efficiency of rRhi o 1 was also tested by passively sensitizing the basophils of non-allergic donor with ten RO positive patient sera. We optimized the amount of allergen required for optimal histamine release to be 30 ng. As illustrated in Fig 4C, 30 ng of Rhi o 1 induced the release of histamine from effector cells pre-sensitized with the serum of ten patients. The amount of histamine released was found within a range from 36% to 49%. Healthy serum and BSA were used as two sets of negative controls, for which histamine release was estimated to be less than 5%.

### Characterization of Rhi o 1 as Aspartyl endopeptidase

**Fig 5:**
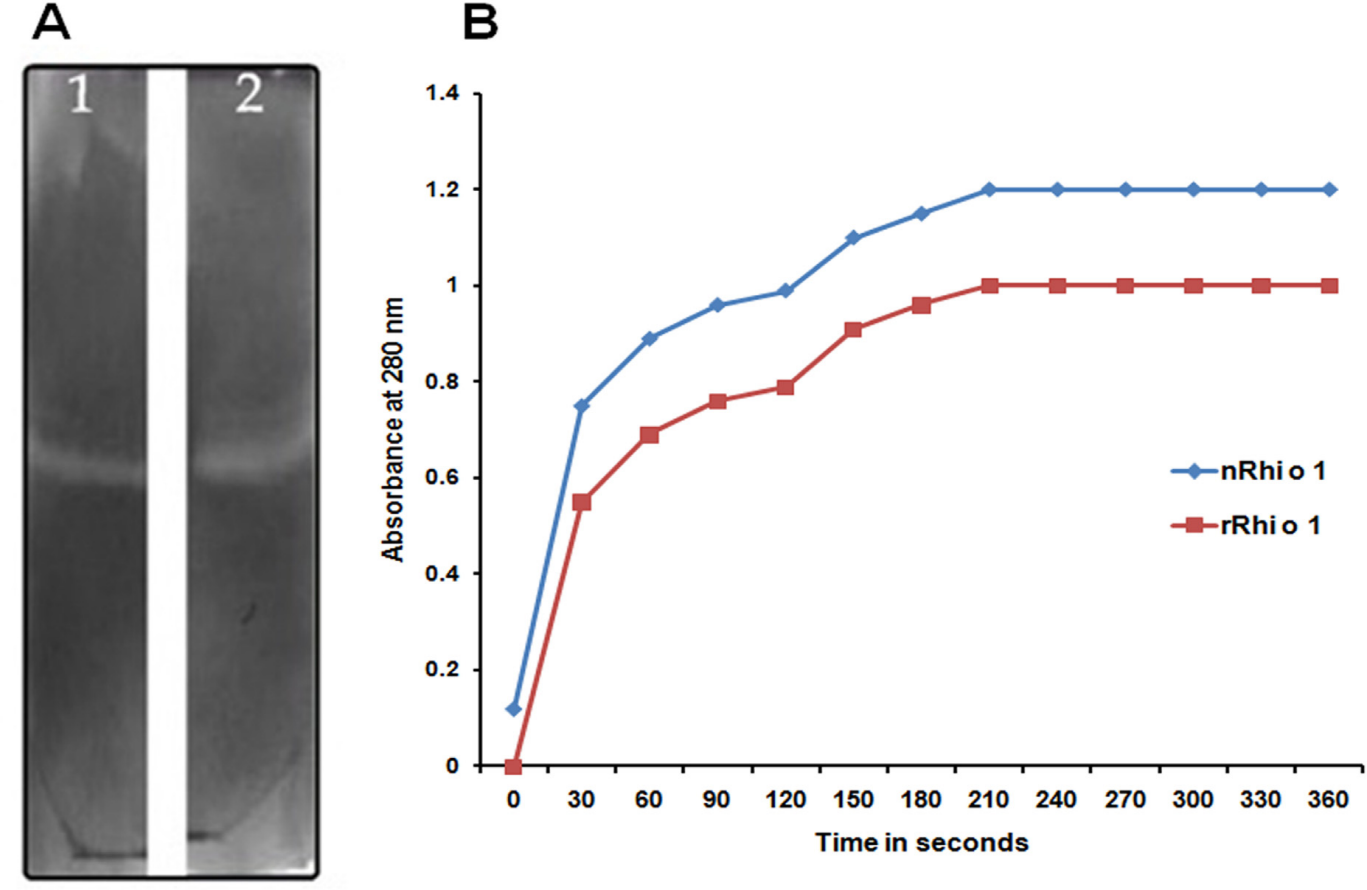
Enzyme assay for characterization of Rhi o 1 as aspartic protease. (A) In-gel: 0.02% Gelatin zymography of purified nRhi o 1 (lane 1) and rRhi o 1 (lane 2) in 7% polyacrylamide gel. Due to protease activity colorless zones appeared at region corresponding to Rhi o 1 position on gel and remaining gel stained blue with CBB-R250 due to presence of gelatin. (B) In-sol: Spectrophotometric assay of aspartic protease with BSA as substrate. 100 nM Rhi o 1 catalyzed degradation of 2% BSA in presence of 50 mM sodium citrate (pH 3.2). After terminating reaction with perchloric acid, precipitated proteins were removed and absorbance of the clear supernatant containing degraded products of BSA was taken at 280 nm. Purified nRhi o 1 (blue line) and rRhi o 1 (red line) displayed time dependent increase in A_280_ of the supernatant.

Gelatine zymography, a gel based assay with gelatine as substrate, was performed to detect the protease activity of the purified native and recombinant Rhi o 1. This assay was based on the fact that the allergen will retain proteinase activity even after electrophoresis and are able to digest the proteins immobilized in polyacrylamide gel. After electrophoresis traces of SDS were removed by repeated washing and incubated in glycine buffer at 37°C to allow renaturation and proteolytic activity to take place. In the gel small colourless areas devoid of Coomassie Brilliant Blue–R250, appeared at the expected molecular weight region i.e. 44 kD corresponding to the position of native and recombinant allergen. Fig 5A represents the gelatine zymography performed with nRhi o 1 and rRhi o 1.

In-sol protease assay of the purified allergens was also performed using BSA as substrate. The allergens were added in the reaction mixture at a concentration at 40 mM and pH- 3.5. The enzymatic activity of aspartyl protease is demonstrated by its ability to catalyse BSA degradation resulting in an increase in A_280_ of the reaction supernatant. Addition of Rhi o 1 caused BSA degradation and concomitant increase in absorbance at 280 nm. As shown in Fig 5B, the rate of protease activity in increasing time course was gradually higher after addition of Rhi o 1. These results clearly indicate that the native and recombinant Rhi o 1 are enzymatically active aspartyl endopetidase.

### CD spectra of nRhi o 1 and rRhi o 1

**Fig 6:**
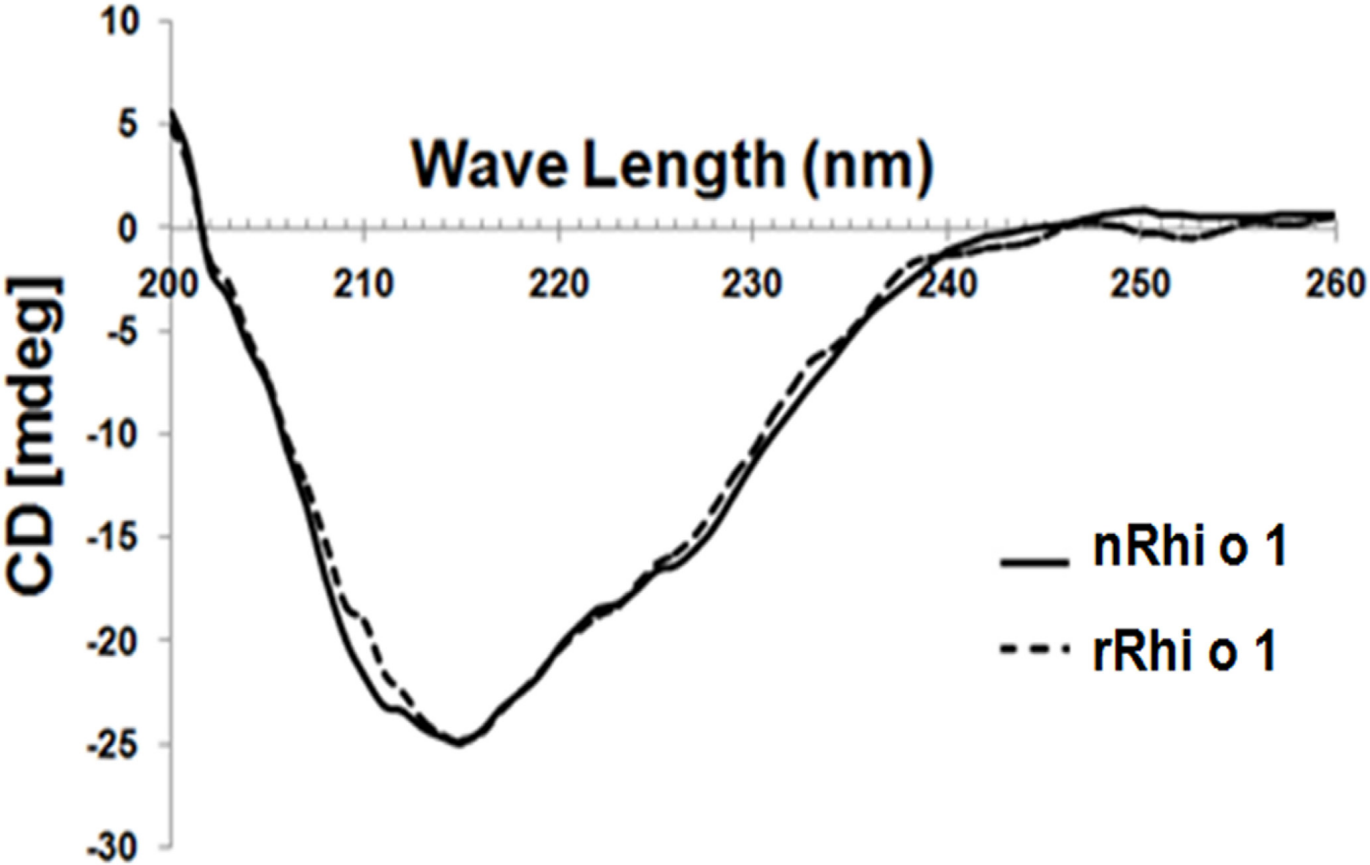
CD spectra of nRhi o 1 (solid line) and rRhi o 1 (discontinuous line) within a range from 200 to 260 nm at constant temperature. Almost similar folding pattern was observed for both the proteins with high content of beat-sheets.

The far-UV spectra (Fig 6) showed higher β-sheet characteristics for Rhi o 1, as minima was obtained at 215 nm. The CD spectrum of the nRhi o 1 was nearly similar to rRhi o 1, indicating that the recombinant allergen has probably adopted the similar folded state.

### Sequence analysis and phylogenetic study on aspartic protease allergens

**Fig 7:**
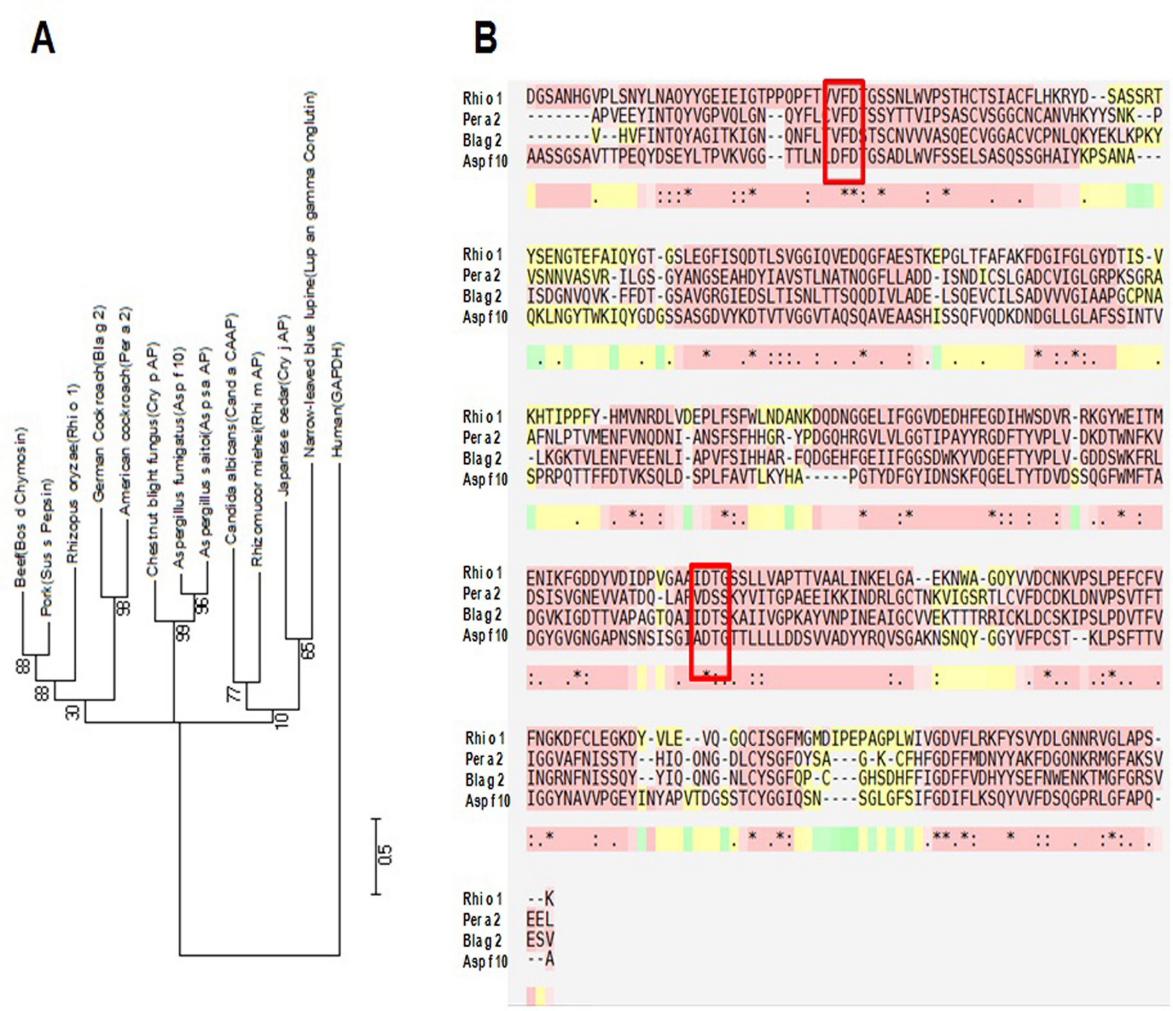
Bioinformatic analysis of aspartic protease allergens. (A) Dendrogram showing evolutionary relationship among twelve aspartic protease allergens (data retrieved from AllFam database) including Rhi o 1 and one unrelated protein (Human Glyceraldehyde phosphate dehydrogenase) as negative control. (B) Multiple sequence alignment of the amino acid sequences of four officially recognized aspartic protease allergens including Rhi o 1 (data retrieved from IUIS allergen database) showing substantial homology. The conserved aspartate residues of the catalytic sites are highlighted in Red box.

Multiple sequence alignment of twelve aspartic protease allergens including Rhi o 1 (see S1 Fig) revealed the presence of fairly conserved residues in the active sites of these proteins. Fig 7A is an un-rooted phylogenetic tree constructed with twelve aspartic protease allergens which clearly showed that Rhi o 1 is evolutionary more close with the animal aspartic proteases whereas other fungal and plant aspartic proteases are relatively distant. Multiple sequence alignment of four well characterized and officially recognized aspartic protease allergens including Rhi o 1 is shown in Fig 7B. From the alignment result it is evident that substantial sequence homology exists among these allergens. Rhi o 1 being a fungal allergen, showed maximum identity of 29% with fungal aspartic protease Asp f 10 followed by 27% identity with each of the two group-2 cockroach allergens Bla g 2 and Per a 2. Two conserved aspartate residues in Rhi o 1 catalytic domain were found to be located in 105^th^ and 290^th^ positions respectively. Alignment result showed that the amino acid sequence of Rhi o 1 is somewhat longer than the others, suggesting the possibility of Rhi o 1 being synthesized as a pro-enzyme with a pro-peptide at its N-terminal. This observation is consistent with the result of N-terminal sequencing result that identified the presence of a 20 amino acid long signal peptide in natural Rhi o 1.

### Rhi o 1 cross reacts to cockroach allergen Bla g 2 with the help of a mAb4C3 recognizable conformational epitope

**Fig 8:**
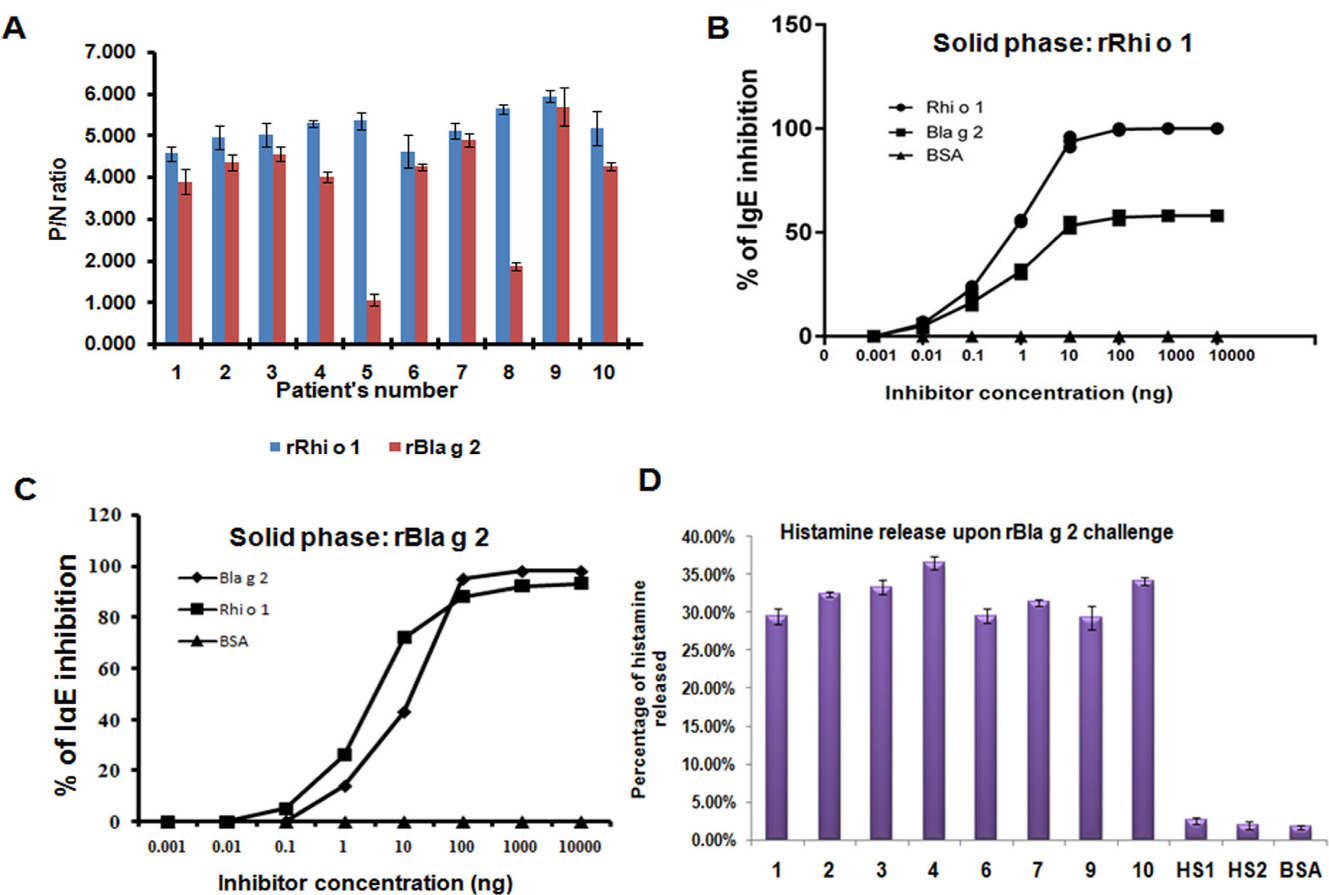
Cross reactivity between Rhi o 1 and Bla g 2. (A) IgE ELISA: rBla g r was tested for its IgE binding to ten Rhi o 1 allergic sera and eight of them were IgE reactive to rBla g 2 (except patient no. 5 and 8) (B) IgE ELISA inhibition: These eight Rhi o 1 positive sera with high anti-Bla g 2 IgE titer were pooled and pre-incubated with increasing concentrations of rBla g 2 (fluid phase inhibitor). rRhi o 1 and BSA instead of rBla g 2 were used as positive (auto inhibition) and negative control (no inhibition) respectively. IgE Binding to plate bound 1 μg of rRhi o 1 was inhibited by rBla g 2 in a dose dependent manner. IC_50_ for rBla g 2 is 20 ng and maximum 58% inhibition was observed with 100 ng rBla g 2. (C) A reciprocal IgE-ELISA inhibition where plate bound 1 μg of rBla g 2 in solid phase was incubated with sera mixed with rRhi o 1 or rBla g 2. In case of rRhi o 1 the IC_50_ is 9 ng and maximum ~93% was achieved at 1000 ng. In case of auto inhibition with rBla g 2 full inhibition was observed. (D) Cross-stimulation experiment: PBMC’s sensitized with eight anti-Rhi o 1 IgE antibody were stimulated with rBla g 2 and histamine release was observed within a range 29% to 36%. No histamine release observed for healthy sera (HS1 and HS2) sensitization and BSA challenge as negative controls.

Inhibition of IgE binding to Rhi o 1 with Bla g 2: Rhi o 1 has fairly good level of sequence homology (27% identity and E-value 4e-31) with another aspartyl protease allergen Bla g 2,which may give rise to common antigenic determinant shared between the two allergens. Here, we tried to check for the presence of any cross-reactivity between these two aspartic proetases. First, we screened ten Rhi o 1 positive sera for IgE binding to rBla g 2 by IgE ELISA. Eight out of ten Rhi o 1 sensitive sera (except patient no. 5 and 8) exhibited significant IgE binding (P/N > 3.5) to rBla g 2 (Fig 8A). These eight sera were selected for subsequent cross reactivity studies and were pooled to perform the immuno-inhibition experiments. ELISA inhibition experiment (Fig 8B) was performed in a panel using rRhi o 1 as plate-bound solid phase and different concentrations of either rBla g 2 or rRhi o 1 as inhibitors. BSA instead of inhibitor was used as negative control. As expected, rRhi o 1 (positive control) showed a dose dependent inhibition of IgE binding to the self protein. Complete auto inhibition (100%) was achieved at a concentration of 60 ng of rRhi o 1 whereas 50% auto inhibition occurred with 5 ng of rRhi o 1 as inhibitor. Next, different concentrations of rBla g 2 were used as fluid phase inhibitor and rRhi o 1 in the solid phase. It was observed that rBla g 2 could partially inhibit specific IgE binding to rRhi o 1. Only 20 ng of Bla g 2 is needed for 50% inhibition and maximum 58% could be achieved with minimum 100 ng of Bla g 2. In case of BSA no inhibition was observed. In a reciprocal experiment illustrated in Fig 8C, rBla g 2 was coated in the ELISA plate as solid phase and probed with the same sera previously used for inhibition assay, mixed separately with serially diluted either rRhi o 1 or rBla g 2. In this assay, 8 ng of rRhi o 1 was found to inhibit 50% IgE binding to rBla g 2 and maximum 93% inhibition was observed at 120 ng concentration. In auto-inhibition, rBla g 2 fully inhibited IgE binding at ~100 ng concentration.

rBla g 2 can cross-stimulate histamine release from PBMC’s sensitized with anti-Rhi o 1 IgE: In order to re-confirm the cross-reactivity between Bla g 2 and Rhi o 1, we performed this assay in addition to the previous cross IgE inhibition assay. As shown in Fig 8D, challenge with rBla g 2 was found to be effective for sufficient histamine release from the PBMC’s sensitized with anti-Rhi o 1 IgE antibodies present in the serum of eight patients. Percentage of histamine released was found to be within a range from 29% to 36% and for healthy sera (as negative control) the value was less than 5%. This result clearly demonstrates that Bla g 2 and Rhi o 1 may share some common IgE epitope(s) responsible for their allergenic activity.

**Fig 9:**
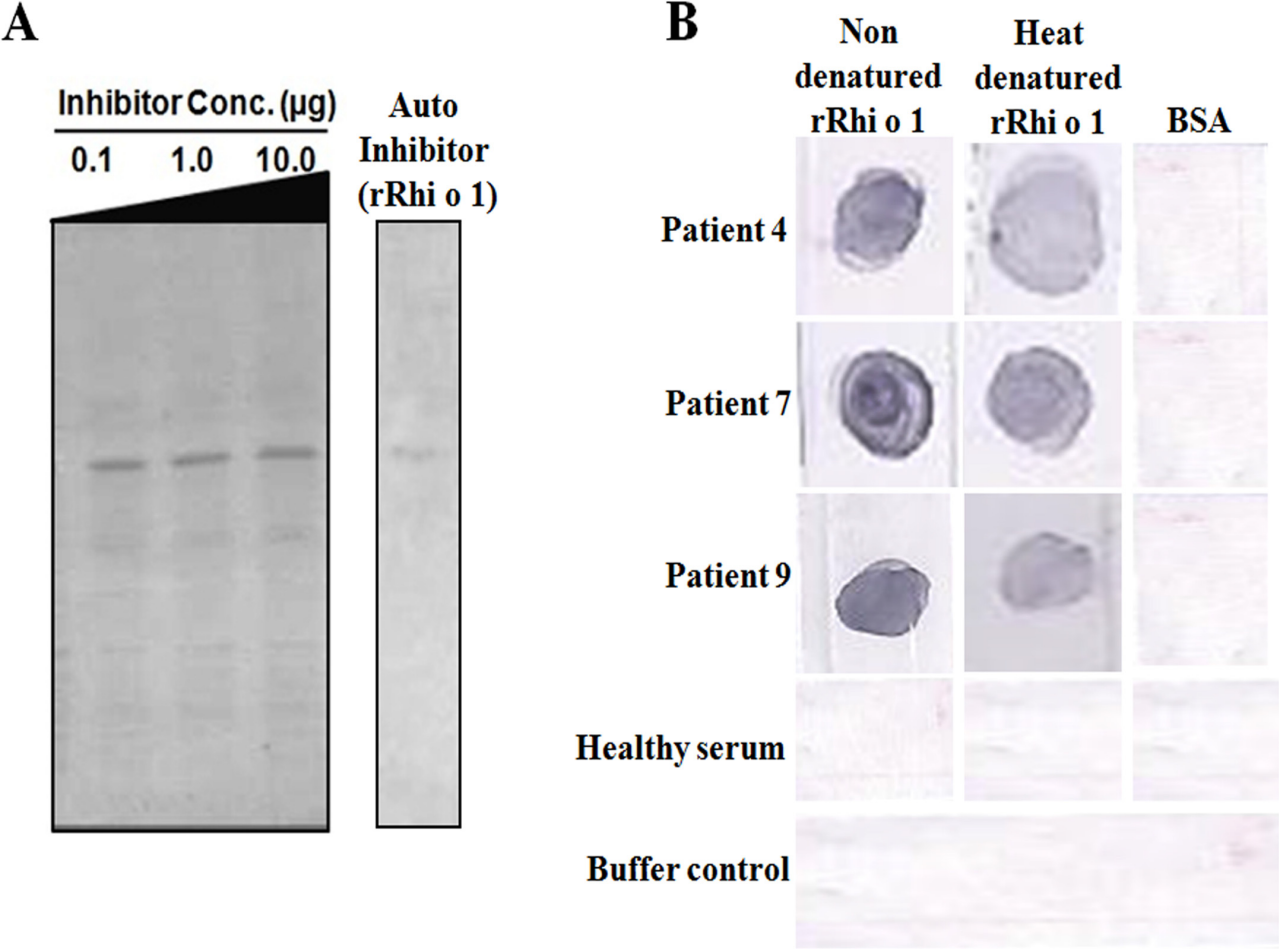
IgE binding to denatured Rhi o 1: (A) IgE blot inhibition: 5 μg rRhi o 1 on PVDF membrane was probed with patient sera containing increasing concentrations (0.1 to 10 μg) of Bla g 2. No visible inhibition of IgE binding to Rhi o 1 was observed in this case, suggesting the role of conformational epitope in cross reactivity. Whatever IgE reactive bands of Rhi o 1 appeared was due to non-cross reactive linear IgE epitopes of Rhi o 1. For auto inhibition positive control, 5μg of self protein (i.e. rRhi o 1) almost fully inhibited IgE binding to membrane bound Rhi o 1. (B) rRhi o 1 was heat denatured and then immediately dotted on PVDF membrane for IgE-blotting with three patient sera. In all the cases heat denatured Rhi o 1 was found to bind IgE as efficiently as the non denatured Rhi o 1. BSA dotted on the same membrane served as negative control. Healthy serum and no serum buffer control are shown in the two lower most panels.

Bla g 2 does not inhibit IgE binding to denatured Rhi o 1: IgE-immunoblot inhibition was also performed to further check the cross reactivity between rRhi o 1 and rBla g 2 under denaturing condition. Unlike the result of ELISA inhibition, it was observed that rBla g 2 was unable to inhibit IgE binding to rRhi o 1 on PVDF membrane transferred from SDS-PAGE. Almost no visible inhibition of IgE binding to rRhi o 1 occurred even at higher concentration (10 μg) of rBla g 2 used for serum IgE saturation. As shown in Fig 9A, the IgE reactive bands of rRhi o 1 were observed at equal intensity in the immunoblots after being confronted with rBla g 2 preincubated sera. On contrary, almost full blot inhibition was observed with 5 μg of auto protein (i.e. Rhi o 1) pre-incubated with sera. In a second experiment, we denatured rRhi o 1 with SDS and heating for 10 mins and this was probed with selected patient sera to test for IgE binding. As illustrated in Fig 9B, the denatured rRhi o 1 was found to bind IgE almost as efficient as the non-denatured rRhi o 1 (used as control).

**Fig 10:**
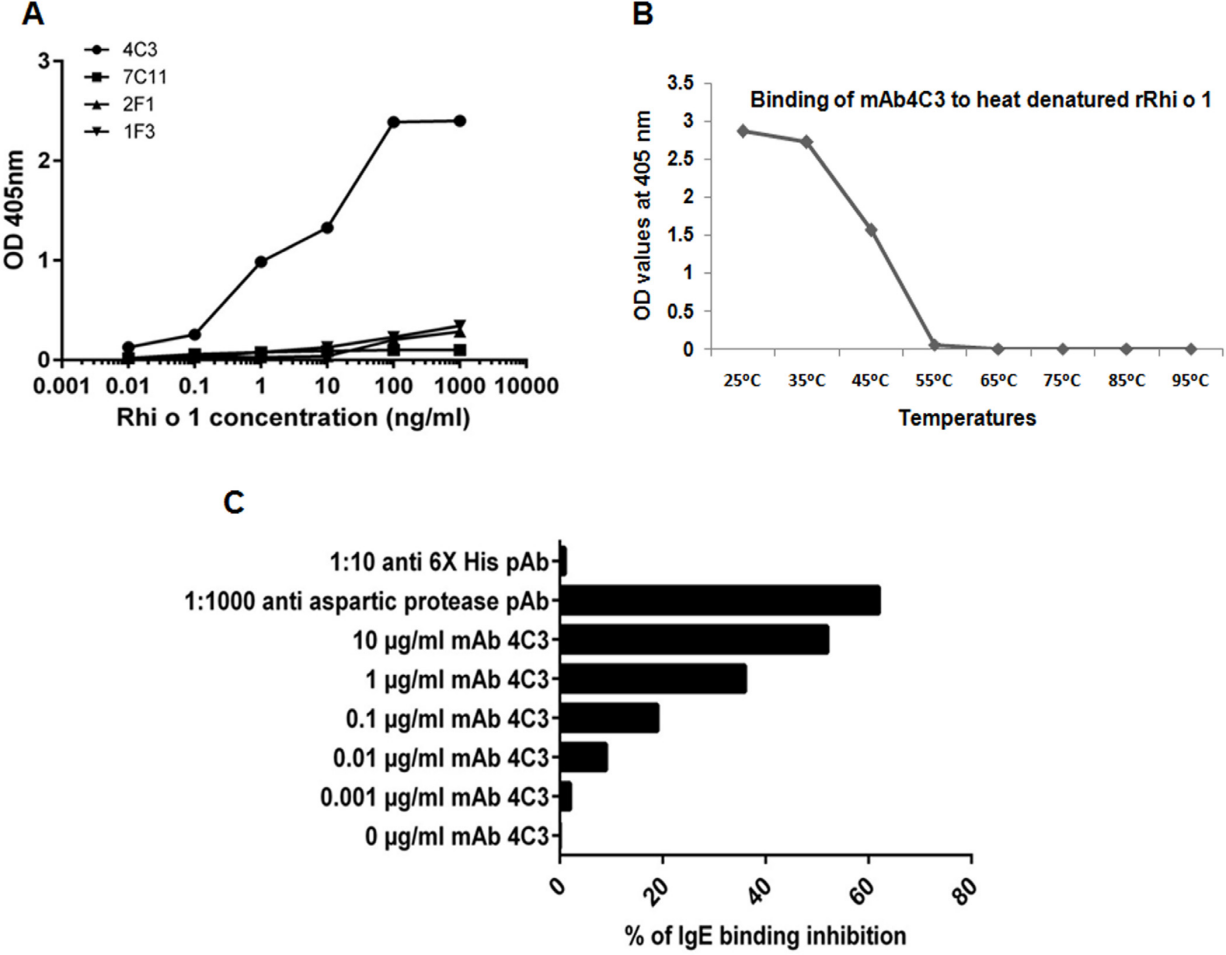
Identification of cross reactive conformational epitope on Rhi o 1 using Bla g 2 specific mAb’s. (A) *Sandwich ELISA*: Increasing concentration of rRhi o 1 was added to four solid phase plate bound anti-Bla g 2 mAb’s 4C3, 2F1, 1F3 and 7C11. Anti-aspartyl protease pAb in rabbit was used as detection Ab and AP tagged anti-rabbit IgG was used as secondary Ab. Only mAb 4C3 displayed dose dependent binding to Rhi o 1 whereas 7C11, 2F1 and 1F3 did not bind to Rhi o 1. (B) *Effect of heat denaturation on mAb4C3 binding*: Binding of mAb4C3 to rRhi o 1 treated at higher temperatures (>50°C) was almost abolished, suggesting the relevance of a conformational epitope in mAb4C3 binding. (C) Inhibition of IgE binding to Rhi o 1 by mAb 4C3: ELISA plates were coated with fixed concentration of rRhi o 1 followed by addition of increasing concentration of 4C3 and cross reactive patient sera. 4C3 showed dose dependent inhibition of IgE binding to Rhi o 1. Maximum 56% inhibition was observed at 10 μg/ml 4C3 concentrations. Anti-aspartyl protease pAb (maximum inhibition) and anti-6x His pAb (no inhibition) instead of 4C3 were used as positive and negative control respectively.

Reactivity of Rhi o 1 with Bla g 2 specific mAb’s 7C11, 2F1, 1F3 and 4C3: In the second step, we tried to identify the possible nature of the cross reactive IgE epitope of Rhi o 1. Four monoclonal antibodies (mAb) have been developed against the surface epitopes of Bla g 2 and interestingly, the epitopes of two of these mAb’s (7C11 and 4C3) are overlapping with the conformational IgE epitopes of the Bla g 2, as shown by earlier workers [14, 15]. Here in the first step, we tested all these four mAb’s for their binding affinity with Rhi o 1. Sandwich ELISA was performed with these four mAbs as capture antibodies. As shown in Fig 10A, out of the four only mAb 4C3 showed significantly strong dose dependent binding with Rhi o 1 with maximum absorbance at 405 nm being 2.389 (SD ±0.087; n = 4). In the second step, the plate bound heat treated rRhi o 1 was incubated with mAb4C3 for binding and it was observed that mAb4C3 displayed decreased binding above 45°C (Fig 10B). At even higher temperatures (55°C and onwards), mAb4C was not able to bind to heat denatured rRhi o 1.This observation led us to conclude that mAb4C3 binding to Rhi o 1 is conformation dependent and is not based on linear peptidic epitopes. In the third step, we tried to investigate whether this mAb4C3 recognizable conformational epitope of Rhi o 1 overlapped with its cross reactive IgE epitope. For this purpose, the plate bound rRhi o 1 was exposed sequentially to patient’s serum IgE and increasing concentrations (from 0.1 to 10 μg/ml) of mAb 4C3. It was observed that, mAb 4C3 exhibited gradually increasing level of inhibition of IgE binding to Rhi o 1 of up to 56%. The anti-aspartyl protease polyclonal antibody produced in rabbit, used as positive control to block the B cell epitopes on Rhi o 1, showed maximum inhibition of IgE antibody binding of up to 62%. On contrary, the negative control i.e. anti-6x His polyclonal antibody, which is specific for the histidine tag of rRhi o 1, showed no IgE inhibition even at dilution as high as 1:10 (Fig 10C). This inhibition assay confirmed an overlap between the IgE epitope and mAb4C3 binding site on Rhi o 1.

### In silico structural study and mapping mAb4C3 epitope on Rhi o 1

**Fig 11:**
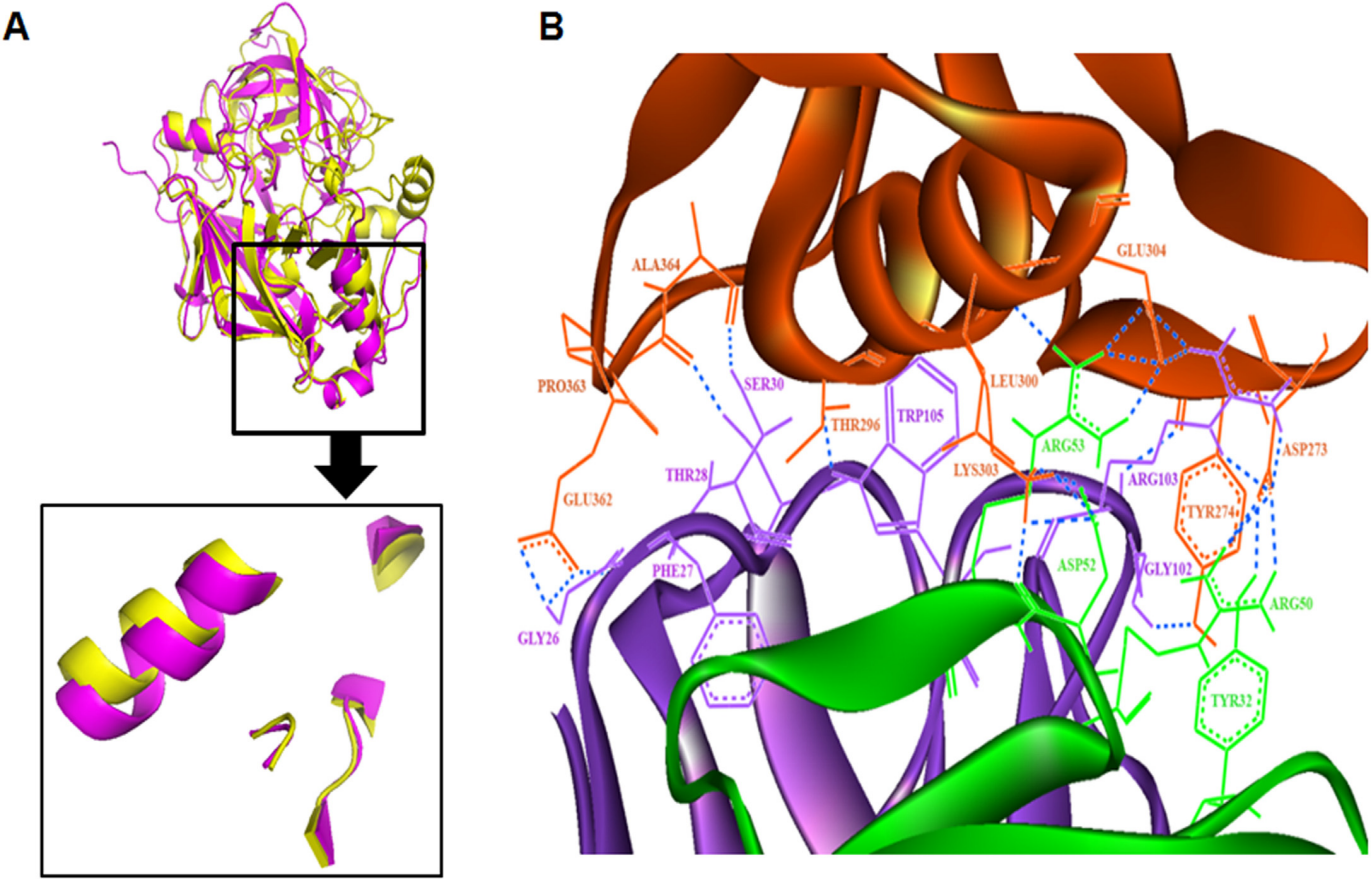
Mapping of cross reactive 4C3 recognizable conformational epitope on Rhi o 1. Superimposition of Rhi o 1 model on Bla g 2 crystal structure allowed identification of common structural elements in the conformational epitope. (A) Alignment of ribbon diagram of Bla g 2 crystal structure (pink) and Rhi o 1 model (yellow) identifies a region on Rhi o 1 (within box) structurally very similar to 4C3 binding region of Bla g 2. (B) Molecular docking of 4C3 crystal structure on Rhi o 1 model: H-bond interaction between the Rhio1 allergen (Brown) docked on mAb 4C3 (Light chain in Green and Heavy chain in Violet colour). The respective residues are labeled and colored accordingly. A Tyrosine^274^ was found to be critical for antibody binding.

Yeast aspartic protease (1DPJ) was used as template for homology modelling of Rhi o 1 which has 61% identity, 75% query coverage and E-value 3e – 156 as revealed by PSI-BLAST. The homology model of Rhi o 1 with full length sequence could not be built due to absence of homologous template with full sequence coverage. The present model was built from 22^nd^ residue to 394^th^ residue of Rhi o 1. This 3D model of Rhi o 1 was found to have a bilobal structure typical for aspartic proteases with two conserved aspartate residues in the substrate binding catalytic core. Alignment of Rhi o 1 model on its template 1DPJ resulted in a backbone R.M.S.D score 0.532 Å. Overall stereochemical quality of the model was evaluated in online SAVES_3 server with 1.5 Å resolution. Main chain conformation of the modelled protein was found to be present in the acceptable region of Ramachandran plot. As estimated in PROCHECK, 81.2% of the residues are within the most favoured regions and 15% were in the allowed regions for this model. Analysis in WHATCHECK package and Verify_3D programme has also considered the model as being fairly good and acceptable. The detail results of overall quality assessment of Rhi o 1 model is described in S2 Fig. Alignment of the atomic coordinates of Bla g 2 with that of the Rhi o 1 resulted in an R.M.S.D value of 1.521 Å. This backbone alignment identified a region on Rhi o 1 model that is structurally very similar to 4C3 recognizable conformational epitope of Bla g 2. This region can be predicted as the possible cross-reactive zone commonly present on Rhi o 1 as well as Bla g 2. The R.M.S.D for only the epitope regions of these two allergens was estimated to be 0.449 Å. The structural elements of the common 4C3 recognizable conformational epitope of Rhi o 1 and Bla g 2 are diagrammatically shown in Fig 11A. This was also verified by molecular docking studies using ClusPro 2.0 server, which produces results based on four types of scoring functions namely Balanced, Electrostatic-favored, Hydrophobic-favored and VdW+Elec. Here we have considered the top ranked antigen-antibody complexes predicted by all these scoring functions and found that the interacting surface area in all the four complexes were nearly similar. For our study we further investigated the top predicted complex from the “Balanced” scoring function as we did not have any prior knowledge about the forces dominating this interaction. From the analysis we found 24 H-bonds interactions between the antigen-antibody complexes (Fig 11B) and as mentioned by Brenke et al., 2012 [33], the phenylalanine, tryptophan and tyrosine residues (Phe27, Trp105 and Tyr32) were present at the interacting surface of the antibody whereas only one tyrosine residue (Try274) was found at the antigen interacting surface.

## Discussion

Prevalence of fungal allergy has been increasing at an alarming rate especially in the urbanized civilization. As compared to pollen or food allergens, however, molecular studies on fungal allergens are really not abundant. *Rhizopus oryzae* is a pathologically important fungus with almost worldwide distribution and was found to trigger respiratory sensitization to atopic individuals. Here, in this study, we describe the molecular and immunological features of an aspartic protease allergen from this indoor mold. It is also the first major allergen reported fromthis mold and a major triggering factor for atopic respiratory diseases such as asthma, rhinitis and often rarely associated with contact skin allergies. This allergen was given the official name‘Rhi o 1’–following the rules of allergen nomenclature proposed by International Union of Immunological Societies (IUIS) allergen nomenclature subcommittee [34].

In our earlier report, a 44 kDa aspartic protease was primarily identified as the major allergen of *R*. *oryzae*. Here, we took an attempt to purify this allergen in native form from the fungal tissue. However, the yield of nRhi o 1 was not sufficient to study further its immunological and biochemical properties. Hence, we utilized the purified nRhi o 1 to determine its amino acid sequence in order to synthetically produce this as a recombinant allergen. The purified nRhi o 1 was found to retain IgE reactivity as immunoscreened with mold allergy patient sera. All the sixteen mold allergy patients with sensitivity against RO, showed IgE reactivity with this allergen. Thus Rhi o 1 can be considered as a ‘major allergen’ of RO. N-terminal sequencing of nRhi o 1 revealed the presence of a 20 amino acid long signal peptide that suggests possible non-cytosolic nature of this allergen. Signal peptides of similar kind are common among certain reported extra-cellular protease allergens [35–37]. Although we found certain N-linked glycosylation sites in Rhi o 1 sequence using certain in-silico server, such as ‘Glycopred’, however,this allergen was found to have no glycosylation as tested by PAS staining.Moreover, the MW of nRhi o 1 in gel seems to be exactly similar to its theoretical MW, as we know for typical glycoproteins, the MW is little bit higher than the theoretical mass. Isoelectrofocusing of nRhi o 1 in 2D gel confirmed the absence of any isoform of this allergen, thus simplifying the use of its recombinant form in clinical diagnosis.

In order to synthetically produce this allergen in large amount and homogenous quality for clinical use, we identified and cloned the cDNA of Rhi o 1 in *E*. *coli*. Bacterially expressed rRhi o 1 was found to retain full allergenicity similar to nRhi o 1, in terms of IgE reactivity as tested by western blot with patient sera and inflammatory mediator release capacity as estimated by histamine assay. Also, the CD spectra of the native and recombinant allergens revealed a similar pattern and thus the similar secondary structure contents. Therefore, the rRhi o 1 can be said as immunologically mimicking its natural counterpart nRhi o 1. The nucleotide sequence and the cDNA clone reported in this study can be used for the development and commercialization of diagnostic kit.

Amino acid sequence revealed the presence of conserved pepsin like aspartic protease catalytic domain in Rhi o 1. Protease activity is a common characteristic feature of many allergens that fosters actin cytoskeletal rearrangements and epithelial desquamation resulting in airway remodelling and increased permeability of lung tissue facilitating easy invasion of the allergen [38, 39]. Another well known aspartic protease allergen is Bla g 2, which was biochemically characterized by earlier workers as a non-functional zinc binding aspartic protease without any enzymatic activity [40]. However, in this study, biochemical assays confirmed Rhi o 1 as a functional aspartic protease active in low pH.

The multiple sequence alignment showed that overall homology exists among the aspartic protease allergens, but long continuous stretch of conserved residues could not be found. It is also clear from the dendrogram that Rhi o 1 in spite of being a fungal allergen is evolutionary more close to aspartic proteases of animal origin. Thus chances of cross reactivity between Rhi o 1 and animal aspartic protease allergens cannot be ruled out, but this cross reactivity is probably not mediated by linear epitopes but the conformational epitope.

In spite of having difference in catalytic property, both Rhi o 1 and Bla g 2 were found to contain a common IgE epitope responsible for their cross reactivity and allergenic activity.In ELISA with anti-Rhi o 1 serum, it was observed that 80% patients had reactivity to rBla g 2. Moreover, the anti-Rhi o 1 specific serum IgE can be partially inhibited from binding to Rhi o 1 by Bla g 2 as observed in ELISA inhibition.This implicated the presence of a common IgE epitope shared by both the allergens. The allergenic activity of these allergens is also influenced by the cross reactive epitope as confirmed by the degranulation assay induced by cross allergen stimulation. During cross allergen stimulation, it was observed that, from Rhi o 1 sensitized PBMC’s, the percentage of histamine release induced by rBla g 2 was to some extent lower than that of rRhi o 1. This could be the possibility of the presence of some non-cross reactive IgE epitopes on Rhi o 1. In a reciprocal ELISA inhibition experiment, it was observed that inhibition of IgE-binding by rRhi o 1 to plate bound Bla g 2 was higher. This observation led us to conclude that in addition to the common IgE epitope, there have been certain other non-cross reactive antigenic determinants on Rhi o 1. These non-cross reactive epitopes may be major and immunodominant for this allergen. Further, it was also found that rBla g 2 can inhibit IgE binding to soluble rRhi o 1 in native conformation. However, in immunoblot, no visible inhibition of IgE binding to Rhi o 1 by Bla g 2 was observed. This observation led us to hypothesize that; here cross reactivity was mediated by a common conformational epitope. Thus in immunoblot under denaturing condition of SDS-PAGE this epitope might have lost its conformation resulting in no inhibition of IgE binding. Whatever IgE reactive bands were visible in immunoblot was mainly contributed by non-cross reactive linear IgE epitopes of Rhi o 1. Furthermore, the efficient IgE binding to heat denatured Rhi o 1 on immuno-dot blot has reconfirmed the presence of certain linear IgE-epitopes on Rhi o 1. There are four murine anti- Bla g 2 mAb’s available and two of which (4C3 and 7C11) are reported as having their epitopes overlapping with the two conformational IgE epitopes of Bla g 2 [14, 15]. Based on this information, we tried to investigate the nature of the cross reactive conformational epitope of Rhi o 1 and whether it is structurally similar to any of these mAb epitopes. It was observed that the mAb4C3 binding can inhibit IgE binding to Rhi o 1, which suggests an overlap between the 4C3 epitope and the corresponding IgE epitope.

This result was further substantiated by an *in silico* structural insight into Rhi o 1 structural model and its comparison with the 4C3 binding site of Bla g 2. High degree of structural resemblances was observed between 4C3 epitope of Bla g 2 and the corresponding aligned regions of Rhi o 1. A Tyrosine residue on Rhi o 1 was found being critical for the interaction as revealed by molecular docking. This was probably the antigenic region on Rhi o 1 responsible for cross reactivity with Bla g 2.

Identification of cross reactivity among allergens provides new insight in utilization of current diagnostic tools and the development of more accurate tests and therapies for allergy [41]. In case of inhalant allergens like Rhi o 1, the discontinuous epitopes are more relevant since these allergens reach the immune system in globular form [42]. However, in the present study it was observed that the denatured Rhi o 1 was efficiently and frequently recognized by the IgE antibody of patient serum and was not at all inhibited from IgE binding by Bla g 2. Also here, two out of ten Rhi o 1 positive sera did not exhibit cross reactivity with Bla g 2. Hence, identification of 4C3 recognizable epitope will not allow unambiguous diagnosis of a genuine sensitization toward the corresponding allergen or the allergen source within a large patient population. It was also pertinent to think that the IgE reactivity of Rhi o 1 was largely contributed by linear IgE epitopes. Thus further studies are warranted to explicate its linear IgE epitopes.

In conclusion, Rhi o 1 is a new addition to the meagre list of recombinant fungal allergens, which is biochemically an active aspartic protease. Rhi o 1, as a major allergen from *Rhizopus oryzae*, triggers IgE mediated sensitization in atopic individuals leading to several allergic diseases. Here, we report the full length cDNA and amino acid sequence of this allergen. The purified recombinant Rhi o 1 will improve the accuracy and specificity of component resolved diagnosis of fungal allergy. Characterization of its conformational epitope, which is responsible for its cross reactivity with the cockroach allergen Bla g 2 has introduced a new notion on the mould–insect cross sensitization and will facilitate designing desensitization therapy. In clinical practice, rRhi o 1 and its epitope information should be considered in the development of allergy vaccines for therapy of patients suffering from mold allergy.

## Supporting Information

S1 Fig Multiple sequence alignment of the amino acid sequences of 12 aspartic protease allergens including Rhi o 1 (data retrieved from AllFam database). From regions with highest sequence homology to regions with lowest sequence homology are shown as red to blue with intermediate shades (green and yellow) represent average homology. Alignment result revealed the presence of fairly conserved residues, especially in the catalytic domain with two conserved aspartate residues marked in red boxes. These 12 allergens were further used for dendrogram analysis.
(TIF)

S2 Fig Overall quality checking of Rhi o 1 model in PROCHECK, WHATCHECK and Verify_3D programmes. (A) Ramachandran plot showing stereo-chemical quality of Rhi o 1 model using PROCHECK server. 81.2% of the residues are within the most favoured regions and 15% were in the allowed regions. (B) Overall quality of ‘Rhi o 1’ model and its deviation from the expected behaviour as assessed in WHATCHECK package. (C) Residue wise plot in Verify3D showing more than 80% residues had scored > = 0.2 in the 3D/1D profile.
(TIF)
