# Supplementary figures and images for: Purification, Cloning and Immuno-Biochemical Characterization of a Fungal Aspartic Protease Allergen Rhi o 1 from the Airborne Mold Rhizopus oryzae
Source: PLoS One. 2015 Dec 16;10(12):e0144547. doi: 10.1371/journal.pone.0144547 (PMC4682942; doi:10.1371/journal.pone.0144547)

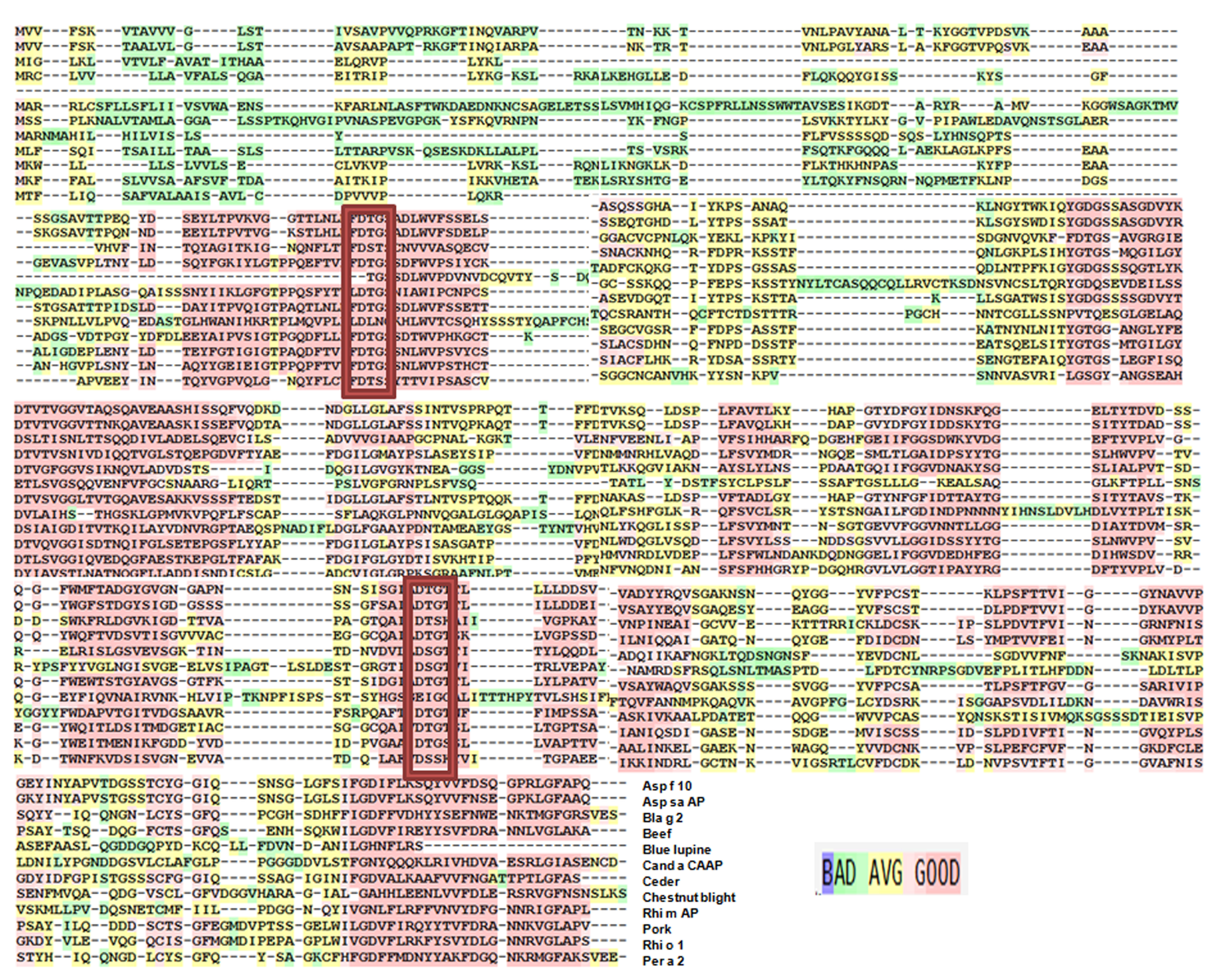

Supplement: S1 Fig — From regions with highest sequence homology to regions with lowest sequence homology are shown as red to blue with intermediate shades (green and yellow) represent average homology. Alignment result revealed the presence of fairly conserved residues, especially in the catalytic domain with two conserved aspartate residues marked in red boxes. These 12 allergens were further used for dendrogram analysis. (TIF) [file pone.0144547.s001.tif]

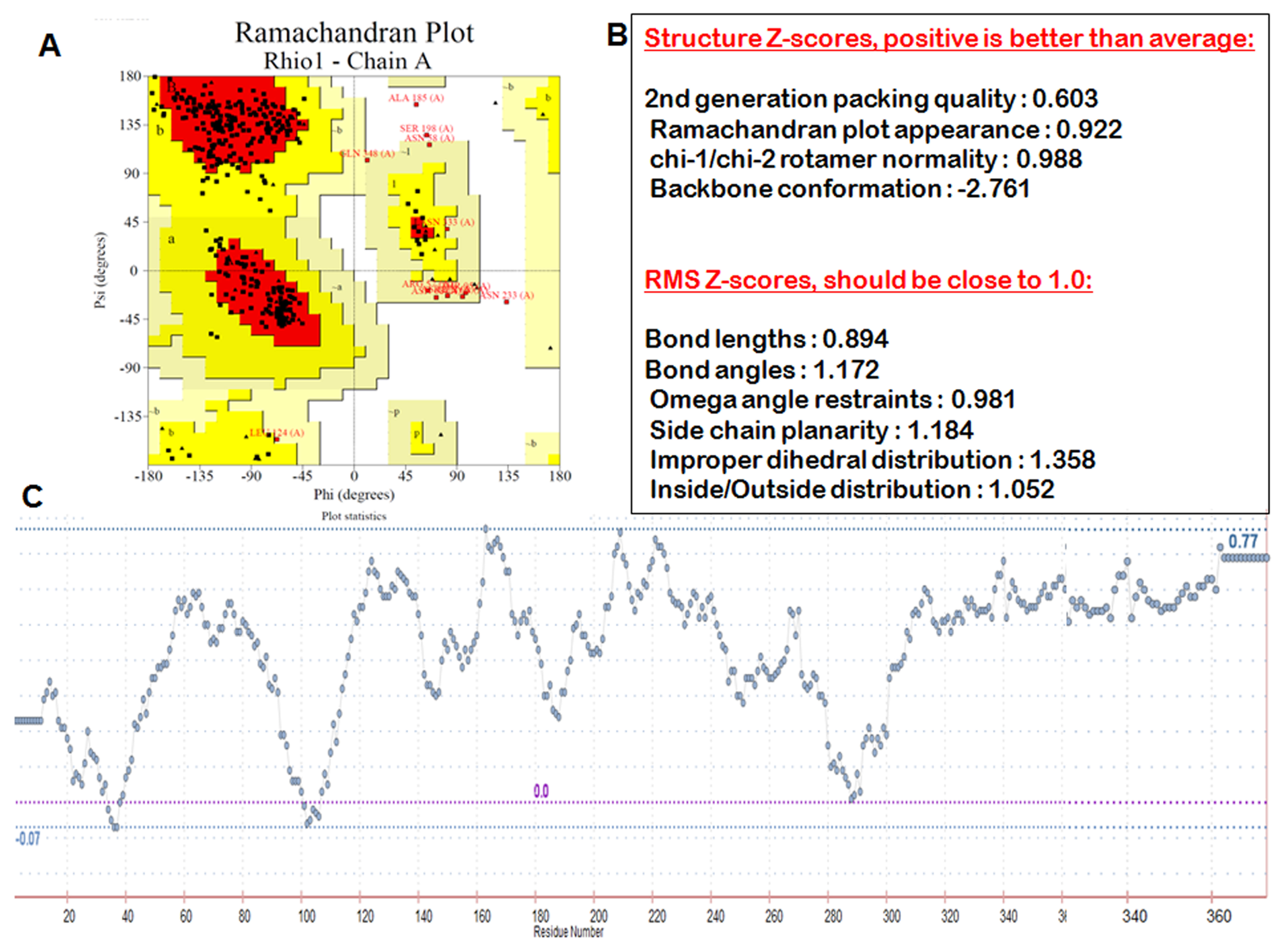

Supplement: S2 Fig — (A) Ramachandran plot showing stereo-chemical quality of Rhi o 1 model using PROCHECK server. 81.2% of the residues are within the most favoured regions and 15% were in the allowed regions. (B) Overall quality of ‘Rhi o 1’ model and its deviation from the expected behaviour as assessed in WHATCHECK package. (C) Residue wise plot in Verify3D showing more than 80% residues had scored > = 0.2 in the 3D/1D profile. (TIF) [file pone.0144547.s002.tif]
